# Genetic and environmental influences on human height from infancy through adulthood at different levels of parental education

**DOI:** 10.1038/s41598-020-64883-8

**Published:** 2020-05-14

**Authors:** Aline Jelenkovic, Reijo Sund, Yoshie Yokoyama, Antti Latvala, Masumi Sugawara, Mami Tanaka, Satoko Matsumoto, Duarte L. Freitas, José Antonio Maia, Ariel Knafo-Noam, David Mankuta, Lior Abramson, Fuling Ji, Feng Ning, Zengchang Pang, Esther Rebato, Kimberly J. Saudino, Tessa L. Cutler, John L. Hopper, Vilhelmina Ullemar, Catarina Almqvist, Patrik K. E. Magnusson, Wendy Cozen, Amie E. Hwang, Thomas M. Mack, Tracy L. Nelson, Keith E. Whitfield, Joohon Sung, Jina Kim, Jooyeon Lee, Sooji Lee, Clare H. Llewellyn, Abigail Fisher, Emanuela Medda, Lorenza Nisticò, Virgilia Toccaceli, Laura A. Baker, Catherine Tuvblad, Robin P. Corley, Brooke M. Huibregtse, Catherine A. Derom, Robert F. Vlietinck, Ruth J. F. Loos, S. Alexandra Burt, Kelly L. Klump, Judy L. Silberg, Hermine H. Maes, Robert F. Krueger, Matt McGue, Shandell Pahlen, Margaret Gatz, David A. Butler, Jennifer R. Harris, Ingunn Brandt, Thomas S. Nilsen, K. Paige Harden, Elliot M. Tucker-Drob, Carol E. Franz, William S. Kremen, Michael J. Lyons, Paul Lichtenstein, Meike Bartels, Catharina E. M. van Beijsterveldt, Gonneke Willemsen, Sevgi Y. Öncel, Fazil Aliev, Hoe-Uk Jeong, Yoon-Mi Hur, Eric Turkheimer, Dorret I. Boomsma, Thorkild I. A. Sørensen, Jaakko Kaprio, Karri Silventoinen

**Affiliations:** 1 Department of Physiology, Faculty of Medicine and Nursing, University of the Basque Country, Bilbao, 48080 Spain; 2 Department of Public Health, University of Helsinki, Helsinki, 00014 Finland; 3 Department of Social Research, University of Helsinki, Helsinki, 00014 Finland; 4 Institute of Clinical Medicine, University of Eastern Finland, Kuopio, 70211 Finland; 5 Department of Public Health Nursing, Osaka City University, Osaka, 545-0051 Japan; 6 Institute for Molecular Medicine FIMM, Helsinki, 00014 Finland; 7 Department of Psychology, Ochanomizu University, Tokyo, 112-8610 Japan; 8 Center for Forensic Mental Health, Chiba University, Chiba, 260-8670 Japan; 9 Institute for Education and Human Development, Ochanomizu University, Tokyo, 112-8610 Japan; 10 Department of Physical Education and Sport, University of Madeira, Funchal, 9020-105 Portugal; 11 CIFI2D, Faculty of Sport, University of Porto, Porto, 4200-450 Portugal; 12 The Hebrew University of Jerusalem, Jerusalem, 91905 Israel; 13 Hadassah Hospital Obstetrics and Gynecology Department, Hebrew University Medical School, Jerusalem, 91905 Israel; 14 Department of Noncommunicable Diseases Prevention, Qingdao Centers for Disease Control and Prevention, Qingdao, 266033 China; 15 Department of Genetics, Physical Anthropology and Animal Physiology, University of the Basque Country UPV/EHU, Bilbao, 48080 Spain; 16 Boston University, Department of Psychological and Brain Sciences, Boston MA, 02215 MA USA; 17 Twins Research Australia, Centre for Epidemiology and Biostatistics, The University of Melbourne, Melbourne, Victoria, 3010 Australia; 18 Department of Epidemiology, School of Public Health, Seoul National University, Seoul, 08826 Korea; 19 Department of Medical Epidemiology and Biostatistics, Karolinska Institutet, Stockholm, 17177 Sweden; 20 Pediatric Allergy and Pulmonology Unit at Astrid Lindgren Children’s Hospital, Karolinska University Hospital, Stockholm, 17176 Sweden; 21 Department of Preventive Medicine, Keck School of Medicine of USC, University of Southern California, Los Angeles, 90089 USA; 22 USC Norris Comprehensive Cancer Center, Los Angeles, 90089 California USA; 23 Department of Health and Exercise Sciences and Colorado School of Public Health, Colorado State University, Colorado, 80523 USA; 24 Department of Psychology, Wayne State University, Detroit, 48202 MI USA; 25 Institute of Health and Environment, Seoul National University, Seoul, 08826 South Korea; 26 Health Behaviour Research Centre, Department of Epidemiology and Public Health, Institute of Epidemiology and Health Care, University College London, London, WC1E 7HB UK; 27 Istituto Superiore di Sanità - Centre for Behavioural Sciences and Mental Health, Rome, 00161 Italy; 28 Department of Psychology, University of Southern California, Los Angeles, CA 90089 USA; 29 School of Law, Psychology and Social Work, Örebro University, Örebro, 701 82 Sweden; 30 Institute for Behavioral Genetics, University of Colorado, Boulder, Colorado 80303 USA; 31 Institute of Behavioral Science, University of Colorado, Boulder, Colorado 80303 USA; 32 Centre of Human Genetics, University Hospitals Leuven, Leuven, B-3000 Belgium; 33 Department of Obstetrics and Gynaecology, Ghent University Hospitals, Ghent, 9820 Belgium; 34 The Charles Bronfman Institute for Personalized Medicine, The Mindich Child Health and Development Institute, Icahn School of Medicine at Mount Sinai, New York, NY 10029-5674 USA; 35 Michigan State University, East Lansing, Michigan 48823 USA; 36 Department of Human and Molecular Genetics, Virginia Institute for Psychiatric and Behavioral Genetics, Virginia Commonwealth University, Richmond, Virginia 23284 USA; 37 Department of Human and Molecular Genetics, Psychiatry & Massey Cancer Center, Virginia Commonwealth University, Richmond, Virginia 23284 USA; 38 Department of Psychology, University of Minnesota, Minneapolis, MN 55455 USA; 39 Center for Economic and Social Research, University of Southern California, Los Angeles, CA 90089 USA; 40 Health and Medicine Division, The National Academies of Sciences, Engineering, and Medicine, Washington, DC 20001 USA; 41 Norwegian Institute of Public Health, Oslo, 0213 Norway; 42 Department of Psychology, University of Texas at Austin, Austin, TX 78712 USA; 43 Department of Psychiatry, University of California, San Diego, CA 92093 USA; 44 VA San Diego Center of Excellence for Stress and Mental Health, La Jolla, CA 92093 USA; 45 Boston University, Department of Psychology, Boston, MA 02215 USA; 46 Department of Biological Psychology, VU University Amsterdam, Amsterdam, 1081 Netherlands; 47 Department of Statistics, Faculty of Arts and Sciences, Kırıkkale University, Kırıkkale, 71450 Turkey; 48 Karabuk University, Faculty of Business, Karabuk, 78050 Turkey; 49 Department of Education, Mokpo National University, Jeonnam, 534-729 South Korea; 50 Department of Psychology, University of Virginia, Charlottesville, VA 22904 USA; 51 Novo Nordisk Foundation Centre for Basic Metabolic Research (Section of Metabolic Genetics), Faculty of Health and Medical Sciences, University of Copenhagen, Copenhagen, 1353 Denmark; 52 Department of Public Health (Section of Epidemiology), Faculty of Health and Medical Sciences, University of Copenhagen, Copenhagen, 1353 Denmark; 53 Osaka University Graduate School of Medicine, Osaka University, Osaka, 565-0871 Japan

**Keywords:** Heritable quantitative trait, Quantitative trait

## Abstract

Genetic factors explain a major proportion of human height variation, but differences in mean stature have also been found between socio-economic categories suggesting a possible effect of environment. By utilizing a classical twin design which allows decomposing the variation of height into genetic and environmental components, we tested the hypothesis that environmental variation in height is greater in offspring of lower educated parents. Twin data from 29 cohorts including 65,978 complete twin pairs with information on height at ages 1 to 69 years and on parental education were pooled allowing the analyses at different ages and in three geographic-cultural regions (Europe, North America and Australia, and East Asia). Parental education mostly showed a positive association with offspring height, with significant associations in mid-childhood and from adolescence onwards. In variance decomposition modeling, the genetic and environmental variance components of height did not show a consistent relation to parental education. A random-effects meta-regression analysis of the aggregate-level data showed a trend towards greater shared environmental variation of height in low parental education families. In conclusion, in our very large dataset from twin cohorts around the globe, these results provide only weak evidence for the study hypothesis.

## Introduction

Since the late 19^th^ and early 20^th^ centuries^1–3^, family, twin and adoption studies have revealed that stature is among the most heritable quantitative traits in humans^4^. Genetic linkage studies have elucidated the location of genetic markers in the genome^5^ and genome-wide association (GWA) studies identified hundreds of loci related to height in different ancestry populations^6–10^. On the other hand, numerous environmental factors in childhood are known to affect growth; disadvantageous environmental conditions may decline the physical growth of children leading to shorter adult height^11–13^. Although nutrition and particularly the lack of dietary protein is the most relevant environmental factor affecting height, childhood diseases, particularly infections, also influence growth^14^. Such environmental exposures are generally shared by siblings to a large extent and would be expected to affect growth rather uniformly within families. These and other biological determinants are in turn related to socio-economic conditions manifesting as socio-economic height differences both between and within populations^13^. Accordingly, social and economic characteristics of childhood families, such as parental education and income, have generally been positively associated with the height of offspring^15–17^.

Twin studies have shown that environmental factors common to co-twins affect variation in height over the lifespan; the percentage of individual differences explained by the common environment was greatest in infancy (up to 50%), decreased over childhood and was generally absent or lower than 20% in adolescence and adulthood^18,19^. The classical twin design^20^ enables variance decomposition into common and unique environmental variance components and a genetic variance component. These components may all vary depending on particular exposures, e.g. exposure to a parental home with parents of lower or higher education. For example, heritability – i.e., the percentage of total variance explained by genetic variance – of height may not, be constant but dependent on the magnitude of environmental variation influencing the phenotype^21^. A poorer household environment may more often than a more affluent one, fail to provide basic necessities and can lead more frequent diseases stunting human growth^13^. This can be reflected in not only to shorter mean height but also higher environmental variation of height in poorer families with siblings being exposed to more similar household environments than non-siblings. On the other hand, in families with a higher socio-economic position, the environment is likely to be more uniformly good with fewer environmental factors restricting growth and thus leading to taller offspring and less environmental variation.

According to the bioecological model, at-risk environments will mask genetic differences between individuals, while enriched environments will amplify genetic differences^22,23^. This leads to the hypothesis, that the heritability of height should increase with higher parental socioeconomic position. To our knowledge, there are no previous studies testing this hypothesis and thus no direct evidence whether the heritability of height differs according to family social background and parental education. Further, such modifying effect of socio-economic characteristics might change over birth cohorts or could be different in males and females, if some cultures would encourage scare resources to be primarily shared with male offspring.

To examine the modification of genetic and environmental variance components by parental education, large datasets collected across a range of strata within society or across different countries are needed. The power to detect such effect was explored by Boomsma and Martin^24^ who concluded that heritability differences between groups of 0.3 or smaller requires large samples. Such information from large datasets was available from 29 twin cohorts participating in the CODATwins (COllaborative project of Development of Anthropometrical measures in Twins) project representing 15 countries from different parts of the world^25^. We utilized this database (i) to test whether parental education modifies the genetic and environmental variation of height in males and females from infancy through adulthood and (ii) to assess whether the possible modification effects vary between different geographic-cultural regions (Europe, North America and Australia, and East Asia).

## Results

Descriptive statistics of height and parental education by age and sex for the pooled data (all cohorts together) are presented in Table 1 (the corresponding statistics by cultural-geographic region are presented in Supplementary table 1). Mean height showed the expected age pattern, and the difference between consecutive age groups was very similar in boys and girls during childhood. The exception was the slight decrease observed at 18 (males) and 20–69 (females) years, which reflects differences in the distribution of different cohorts within each age group. Mean height was generally tallest in Europe, somewhat shorter in North America and Australia and shortest in East Asia in both males and females. Paternal and maternal education generally decreased with age, which reflects the increasing education over birth cohorts since parents of younger twins were, on average, born later as compared to parents of older twins. Parental education was virtually identical for male and female twins during childhood and slightly greater in females from late adolescence. Parental education was generally lowest in Europe, reflecting that European twin cohorts were older than North American and Australian and East Asian cohorts (Supplementary table 1).

**Table 1:** Number of measurements, means and standard deviations (SD) of height and parental education by age and sex.

Age	Males							Females						
		Height (cm)		Paternal education (years)		Maternal education (years)			Height (cm)		Paternal education (years)		Maternal education (years)	
	N	Mean	SD	Mean	SD	Mean	SD	N	Mean	SD	Mean	SD	Mean	SD
1	13155	75.0	3.60	13.85	2.58	13.79	2.30	13631	73.5	3.67	13.86	2.61	13.82	2.31
2	10912	87.5	4.02	13.97	2.69	13.98	2.40	10930	86.2	4.15	13.91	2.75	13.99	2.43
3	10541	96.8	4.43	14.17	2.73	14.20	2.48	11087	95.8	4.50	14.13	2.75	14.20	2.50
4	3307	101.8	5.79	14.74	3.64	15.34	3.40	3327	100.6	5.72	14.74	3.68	15.45	3.38
5	6269	111.8	6.12	14.28	2.83	14.38	2.60	6341	111.0	6.28	14.27	2.84	14.31	2.52
6	1726	114.5	7.14	14.99	3.23	15.13	3.07	1796	113.8	6.64	15.04	3.29	15.22	3.09
7	6852	125.6	6.71	14.31	2.63	14.26	2.42	7228	124.9	6.55	14.31	2.68	14.22	2.45
8	4153	129.4	6.43	14.32	2.87	14.41	2.78	4261	128.4	6.57	14.32	2.93	14.35	2.77
9	3310	134.8	7.35	14.43	3.23	14.66	3.14	3266	133.9	7.47	14.55	3.28	14.77	3.13
10	6776	142.1	7.15	14.25	2.70	14.12	2.55	7136	141.5	7.35	14.21	2.62	14.00	2.42
11	3751	144.9	7.29	12.87	4.02	13.32	3.67	3779	145.3	7.73	12.92	4.06	13.36	3.67
12	6522	152.9	8.06	13.82	3.12	13.75	2.75	6750	154.0	8.10	13.83	3.17	13.74	2.77
13	2834	158.4	9.21	14.23	3.05	14.33	2.84	3102	157.8	7.67	14.23	2.96	14.13	2.78
14	4860	165.8	8.99	12.80	4.01	13.20	3.63	5402	162.2	6.93	12.84	3.96	13.29	3.52
15	2753	172.2	8.60	14.27	3.04	14.21	2.88	3027	164.4	7.40	14.25	2.97	14.18	2.70
16	3487	175.3	7.85	13.19	3.33	13.04	3.18	3979	164.7	6.82	13.10	3.27	13.04	3.11
17	4679	177.6	7.44	12.80	3.58	12.94	3.36	5187	165.7	6.88	12.97	3.50	13.06	3.20
18	3488	177.1	7.68	11.81	4.07	12.14	3.58	3230	165.8	7.21	12.44	3.83	12.61	3.45
19	2073	178.2	7.57	12.39	3.31	12.30	2.95	2547	165.7	7.19	13.13	3.08	12.90	2.84
20–69	25951	178.4	7.16	11.82	3.66	11.89	3.20	31205	164.5	6.76	12.25	3.54	12.15	3.18

Names list of the participating twin cohorts in this study: one cohort from Australia (Australian Twin Registry), five cohorts from East Asia (Korean Twin-Family Register, Ochanomizu University Twin Project, Qingdao Twin Registry of Children, South Korea Twin Registry, West Japan Twins and Higher Order Multiple Births Registry), 11 cohorts from Europe (Child and Adolescent Twin Study in Sweden, East Flanders Prospective Twin Survey, FinnTwin12, FinnTwin16, Gemini, Italian Twin Registry, Norwegian Twin Registry, Portugal Twin Cohort, TCHAD-study, Turkish Twin Study, Young Netherlands Twin Registry), one cohort from Middle East (Longitudinal Israeli Study of Twins) and 11 cohorts from North America (Boston University Twin Project, California Twin Program, Carolina African American Twin Study of Aging, Colorado Twin Registry, Michigan Twins Project, Mid Atlantic Twin Registry, Minnesota Twin Registry, NAS-NRC Study, University of Southern California Twin Study, Texas Twin Project, Vietnam Era Twin Registry).

**Figure 1:**
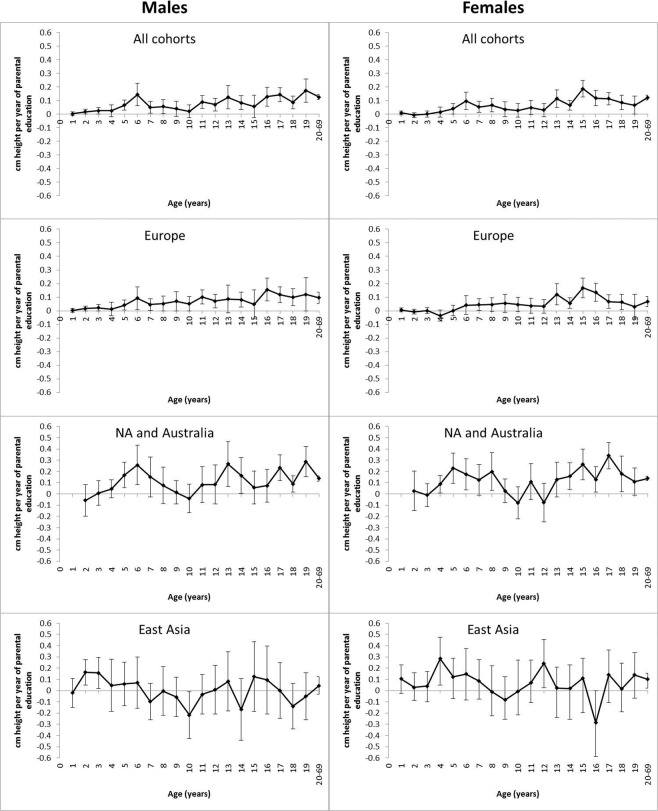
Mean height modification effects of parental education with 95% confidence intervals from 1 until 20–69 years of age by sex and geographic-cultural region.

The associations between parental education (i.e., combined maternal and paternal) and offspring height, i.e. height difference in cm by one year difference of parental education, are presented in Fig. 1. From around age 5 years, parental education showed a generally positive association with offspring height; the pattern was similar in males and females, with significant associations in mid-childhood and from adolescence onwards. Regarding the geographic-cultural regions – which approximate ethnicity in the present study – the pattern in Europe was similar to that observed for the whole data set because it represents a large fraction of the total sample. In North America and Australia, the associations between parental education and offspring height were stronger than in Europe in some age groups, particularly in mid-childhood. In East Asia, the associations generally varied around zero and were not statistically significant. In North America and Australia and East Asia, the 95% confidence intervals (CIs) were, however, much broader than in Europe because of the smaller sample sizes.

**Figure 2:**
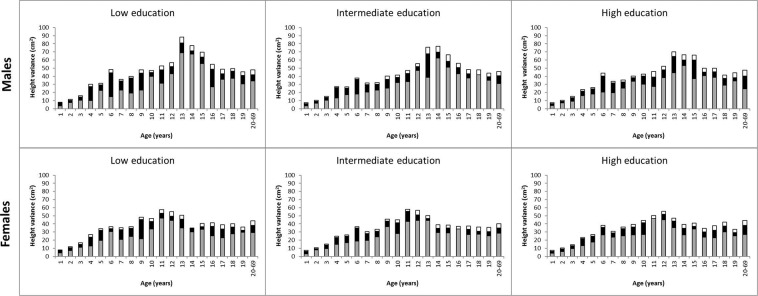
Additive genetic (grey), shared environmental (black) and unique environmental (white) variances of height from 1 until 20–69 years of age by sex and parental education in all cohorts.

**Figure 3:**
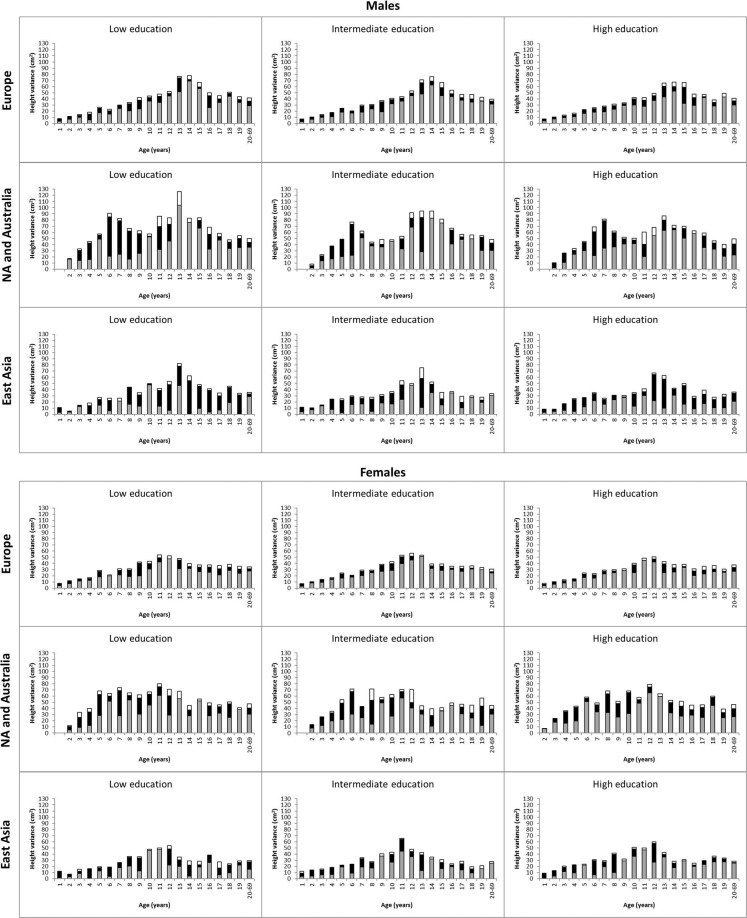
Additive genetic (grey), shared environmental (black) and unique environmental (white) variances of height from 1 until 20–69 years of age by sex, parental education and geographic-cultural region.

The total variance of height decomposed into additive genetic, shared environmental and unique environmental components in the three categories of parental education is shown in Fig. 2 (the estimates with 95% CIs are available in Supplementary Table 2). The total height variation was slightly greater in the lower than in the higher parental education level in some age-by-sex groups, but no consistent relation emerged by educational categories over ages. From age 13 years onwards, the total height variance was generally greater in males than in females. As indicated by overlapping CIs, genetic and environmental variances did not show any distinct relation across parental education categories from infancy through adulthood; the relative proportion of genetic and environmental variances did not show any relation either (Supplementary Table 3). Next, univariate variance decomposition modeling for height was carried out separately in the three geographic-cultural regions (Fig. 3 and Supplementary Tables 4 and 5). The total variance of height was greatest in North America and Australia and lowest in East Asia, but no distinct relation in the variance components (both total estimates and relative proportion) across the parental education levels emerged (seen as overlapping CIs). In East Asia, possibly due to the smaller sample sizes, the magnitude of the variance components between the educational categories varied more than in the other two geographic-cultural regions.

Finally, we ran a random-effects meta-regression analysis of raw variance components of height (pooling all age groups and geographic-cultural regions together). The results showed some significant differences between the middle and low parental education categories (Table 2), when looking at the confidence intervals. In comparison with low parental education, for middle education shared environmental (c^2^) component of height was significantly smaller in males and in both sexes together. The point estimates for the other sex and variance components groups followed the same direction, but were not significant. Given the number of comparisons, we should be very careful in a substantive interpretation of these findings. Standardized variance components models gave very similar results (Supplementary Table 6).

**Table 2:** Regression coefficients from meta-regression analyses of the aggregate-level data of raw variance components of height by parental education (reference category: low parental education).

	Intermediate parental education	High parental education
**Males**		
a^2^	2.13 (−1.48, 5.74)	−0.01 (−3.66, 3.63)
c^2^	−3.27 (−6.31, −0.23)	−1.66 (−4.79, 1.46)
e^2^	−0.15 (−0.68, 0.38)	−0.26 (−0.79, 0.27)
**Females**		
a^2^	1.05 (−0.81, 2.91)	0.26 (−1.65, 2.18)
c^2^	−1.69 (−3.64, 0.26)	−1.57 (−3.55, 0.42)
e^2^	−0.23 (−0.67, 0.21)	−0.36 (−0.80, 0.08)
**Both sexes**		
a^2^	1.46 (−0.76, 3.69)	0.67 (−1.58, 2.92)
c^2^	−2.30 (−4.05, −0.55)	−1.58 (−3.37, 0.21)
e^2^	−0.21 (−0.54, 0.13)	−0.31 (−0.65, 0.02)

(): 95% Confidence Intervals.

## Discussion

Questions about the modification of genetic and environmental variance components require very large and genetically informative data sets. Our large twin study pooling data for 65,978 complete twin pairs from 29 cohorts from 15 countries established that for human height there is a high and consistent heritability across parental education levels. The same result, i.e. similar genetic and environmental variances of height across parental education levels, was found in different geographic-cultural regions having different mean stature.

The meta-regression analysis also failed to provide substantial evidence for the study hypothesis that shared environmental variation of height tends to be greater in low parental education families; the evidence is weak considering the size of the dataset when pooling all data together. In a previous study from the CODATwins database, we found that there was no decrease in the environmental variance of adult height over the birth cohorts from the late 19^th^ century to the late 20^th^ century, nor any clear secular changes in the heritability^18^. Therefore, using two very different approaches –i.e., indirect information on the increasing standard of living over 100 years and the direct measures of socio-economic position of childhood family– we established that there is no or very little evidence of greater shared environmental variation in height in disadvantageous environments.

The offspring of better educated parents were generally taller, particularly in mid-childhood and from adolescence onwards, than those whose parents had lower education. Our findings for average height are in agreement with several population based studies showing a positive association between parents’ education and offspring height^15,17,26,27^. In a Chinese study, childhood height was also related to grandparents’ education, suggesting that socioeconomic conditions of current and previous generations may affect height^28^. In some societies, children from families with lower socioeconomic status (SES) may still have, on average, poorer diets and be more severely affected by infections than those from families with higher SES^13–15^. Comparison between geographic-cultural regions showed that parental education was more strongly related to height in North America and Australia than in Europe, which may reflect larger social inequalities in the former.

In the families of lower SES, environmental effects (e.g. malnutrition) on height may restrict individuals from reaching their genetic potential, leading to shorter stature. It is likely that there are differences in these environmental factors between low SES families; in high SES families, in contrast, the environment securing optimal growth is likely to be more homogeneous. These environmental influences would result in more between than within family variation in lower SES families, which according to the bioecological model is expected to increase shared environmental variation leading to lower heritability of height in lower as compared with higher SES families. It is thus interesting that even when we found the expected differences in mean height between families of high and low parental education, only very weak differences in genetic or environmental variances or in the heritability estimates of height were observed. It is theoretically possible that environmental factors affecting growth are so uniformly distributed in lower SES families that there is no variance of height explained by these environmental factors and thus the influence is not seen as shared environmental variation. However, we do not find this very likely since it would mean that families with high and low parental education form two distinctive but internally very homogenous groups. Further, this should be the case in all three cultural-geographic regions.

Finally, it is possible that the differences in height between the families of high and low parental education are not because of a causal effect of poorer living conditions on height but reflect genetic height differences. A study of children born in the 1990s found that higher education mothers had taller sons and daughters and that these differences in offspring height were fully explained by parental height^26^. This can be explained also by inheritance of socio-economic factors and not only genetic factors affecting height. However, there is also direct evidence on a modest genetic correlation (r = 0.13) between education and height based on linkage disequilibrium score regression analyses^29^. Thus, a not unreasonable hypothesis is that genetic variance of height can also differ by parental educational level. Such hypotheses will be testable in future studies, with the increasing availability of large genotyped cohorts (e.g.^30^).

The present study has several strengths. First of all, our large multinational database of twin cohorts, with data on parental education and height over childhood and adulthood, allows a comprehensive research of the genetic and environmental influences on individual height differences across parental education categories over lifespan in different cultural-geographic regions. We had sufficient statistical power to address these questions. The individual-based data, in comparison to literature based meta-analyses, provide important advantages such as better opportunities for statistical modeling and lack of publication bias. However, our study also has limitations. Ethnic-cultural groups are differently represented and the greatest proportion of the database is formed by Caucasian populations following Westernized lifestyles. In addition, most of the height measures were self-reported^31^, which increases measurement error and thus may bias our results toward greater estimates of unique environmental effects. However, this is not likely to explain the main result, i.e., relatively similar genetic and environmental variances of height across the categories of parental educational attainment. Also when pooling the estimates of variance components from different ages, we could not adjust the SEs by multiple observations at different ages, and thus, the 95% CIs are likely to be too narrow. Therefore, the main emphasis should be on the age-specific results, where only one observation from each individual is used.

In conclusion, there is no solid evidence that lower parental education is related to greater environmental variation in offspring height from infancy through adulthood. Thus, our findings indicate that the heritability estimates of height are quite uniform across parental education levels in spite of differences in mean height.

## Materials and methods

### Sample

This study is performed with data from the CODATwins project, which was planned to pool information on height and weight data from all twin projects in the world^31^. Additional information on paternal and maternal education was available for 29 twin cohorts from 15 countries. The participating twin cohorts are listed in Table 1 (footnote) and were described in detail elsewhere^25,31^.

In the original database, there were 137,867 twin individuals with a total of 311,087 height measurements at ages 1–69 years. Age was classified to single-year age groups from age 1 to 19 years (e.g. age 1 includes 0.5–1.5 years range) and one unique adult age group (20–69 years); height measures at ages ≥70 years were excluded because individuals in old age are more prone to develop osteoporosis leading to shorter height^32^. Outliers and implausible values were checked by visual inspection for each age and sex group and removed (0.1% of the measurements) to obtain an approximately normal distribution, resulting in 310,736 measurements. To confirm that all analyses are based on independent observations, we selected one height measure per individual in each age group by keeping the measurement at the youngest age (removing <10% of the measurements) resulting in 282,176 height measurements from 137,574 twin individuals. After excluding twins without data on their co-twins, we had 264,610 height measurements (132,305 paired height measurements; 38% monozygotic (MZ), 34% same- sex dizygotic (SSDZ) and 28% opposite-sex dizygotic (OSDZ) twin pairs) from 65,978 complete twin pairs (the number of observations by age and twin cohort is available on request). The different educational classifications used in the surveys were transformed as educational years by using the mean level of educational years in each category as described in detail elsewhere^25^.

In order to analyze possible differences in the genetic and environmental contribution on height across geographical-cultural regions, the cohorts were grouped in three regions: Europe (10 cohorts), North America and Australia (12 cohorts) and East Asia (5 cohorts) with 88,632, 34,087 and 8,873 paired height measurements, respectively. Two cohorts (Israel and Turkey) were not included in these sub-analyses by geographic-cultural region because the populations in these countries differ genetically from European populations^33^, and the data were too sparse to study these cohorts separately. The same classification was used also in our previous studies on the genetics of height in childhood^19^ and adulthood^18^ based on the CODATwins database.

All participants were volunteers and they or their parents/legal guardians gave informed consent when participating in their original study. Only a limited set of observational variables and anonymized data were delivered to the data management center at University of Helsinki. The pooled analysis was approved by the ethical committee of Department of Public Health, University of Helsinki, and the methods were carried out in accordance with the approved guidelines.

### Statistical analyses

Statistical analyses were conducted using Stata statistical software (version 14.0; StataCorp, College Station, Texas, USA). First, all height measurements were adjusted for exact age and twin cohort within each age and sex group using linear regression model (height was used as the dependent variable and exact age and twin cohort as independent variables) and the resulting residuals were used as the outcome variable in the further statistical modeling. Twin cohorts were numbered as a nominal level variable in the regression analyses (i.e., a separate dummy variable was created for each twin cohort). Since paternal and maternal education (ranging from 0 to 30 years) may be differently associated with offspring birth year, we adjusted maternal and paternal education separately for twin cohort and birth year of their twin children (used as a proxy indicator for the birth years of parents) by fitting a regression model (maternal or paternal education was used as the dependent variable and twin cohort and birth year of their twin children as independent variables). Thus, the residuals indicate how much shorter or longer the parental education duration is as compared with that of the average person having a certain birth year in each twin cohort. These regression residuals were then summed up to get combined parental education and divided into three SD-based categories (<−0.5, −0.5 to +0.5, > +0.5), indicating low, intermediate and high parental education (31%, 40% and 29% of the observations, respectively).

We first studied the association between height and parental education separately for each age and sex group in all cohorts together as well as by the geographic-cultural regions. Linear regression models were used with parental education as the explanatory variable and height residuals as the outcome. The associations were adjusted for zygosity because of slight differences in height^34^ and parental education between MZ and DZ twins^25^. The non-independence within twin pairs was taken into account by using the cluster-option available in Stata^35^. This option takes into account that twin pairs rather than independent individuals are sampled and accordingly corrects the standard errors to be larger because of the less informative sample design.

To estimate genetic and environmental influences on the variation of height, we employed classic twin modeling based on linear structural equations^36^. MZ twins share the same genomic sequence, whereas DZ twins share, on average, 50% of their genes identical-by-descent. On this basis, it is possible to decompose the total variance of height into variance due to additive genetic effects (A: correlated 1.0 for MZ and 0.5 for DZ pairs), dominance genetic effects (D: 1.0 for MZ and 0.25 for DZ pairs), common (shared) environmental effects (C: by definition, correlated 1.0 for MZ and DZ pairs) and unique (non-shared) environmental effects (E: by definition, uncorrelated in MZ and DZ pairs). As in our previous studies in children^18^ and adults^17^, we found evidence of shared environmental variation but no evidence of dominance genetic variation in height. Thus, we used the additive genetic/shared environment/unique environment model in the analyses. Models were fitted separately for each parental education category by age and sex groups. A clear sex-specific genetic effect for height was found in childhood^19^ and adulthood^18^, and thus it was included in all models allowing the opposite-sex DZ genetic correlation to be lower than the 0.5. Because DZ twins were slightly taller than MZ twins from infancy to adulthood^34^, different means for MZ and DZ twins were allowed. All genetic models were fitted by the OpenMx package (version 2.0.1) in the R statistical platform^31^ using the maximum likelihood method.

In order to test whether variance components of height were significantly different between parental education categories, we ran a random-effects meta-regression analysis of the aggregate-level data of raw variance components. Adjustments were carried out for geographic-cultural regions and age categories, and models were run separately by sex and for both sexes together. However, it should be noted that in these analyses the SEs are not corrected for multiple observations and consequently the 95% CI are likely to be somewhat too narrow, possibly leading to a spurious support of the original hypothesis.

## Supplementary information

Supplementary Tables.

## Data Availability

The data used in this study is owned by the third parties (the individual twin cohorts) and made available to us in condition that they will be used only in this meta-analysis. For this reason, we do not have legal rights to re- deliver the data or to provide it to other third parties without permissions from the data owners. In order to replicate the results, each researcher need to apply the data set from each individual twin cohort owners and to harmonize the data as a metafile.
