# Supplementary material for: Genetic and environmental influences on human height from infancy through adulthood at different levels of parental education
Source: Sci Rep. 2020 May 14;10:7974. doi: 10.1038/s41598-020-64883-8 (PMC7224277; doi:10.1038/s41598-020-64883-8)
Supplement: Supplementary file 1 — Supplementary Tables. [file 41598_2020_64883_MOESM1_ESM.docx]

Genetic and environmental influences on human height from infancy through adulthood at different levels of parental education

Aline Jelenkovic* (1) (2), Reijo Sund (3) (4), Yoshie Yokoyama (5), Antti Latvala (3) (6), Masumi Sugawara (7), Mami Tanaka (8), Satoko Matsumoto (9), Duarte L Freitas (10), José Antonio Maia (11), Ariel Knafo-Noam (12), David Mankuta (13), Lior Abramson (12), Fuling Ji (14), Feng Ning (14), Zengchang Pang (14), Esther Rebato (15), Kimberly J Saudino (16), Tessa L Cutler (17), John L Hopper (17) (18), Vilhelmina Ullemar (19), Catarina Almqvist (19) (20), Patrik KE Magnusson (19), Wendy Cozen (21) (22), Amie E Hwang (21) (22), Thomas M Mack (21) (22), Tracy L Nelson (23), Keith E Whitfield (24), Joohon Sung (18) (25), Jina Kim (18), Jooyeon Lee (18), Sooji Lee (18), Clare H Llewellyn (26), Abigail Fisher (26), Emanuela Medda (27), Lorenza Nisticò (27), Virgilia Toccaceli (27), Laura A Baker (28), Catherine Tuvblad (28) (29), Robin P Corley (30), Brooke M Huibregtse (31), Catherine A Derom (32) (33), Robert F Vlietinck (32), Ruth JF Loos (34), S Alexandra Burt (35), Kelly L Klump (35), Judy L Silberg (36), Hermine H Maes (37), Robert F Krueger (38), Matt McGue (38), Shandell Pahlen (38), Margaret Gatz (19) (39), David A Butler (40), Jennifer R Harris (41), Ingunn Brandt (41), Thomas S Nilsen (41), K Paige Harden (42), Elliot M Tucker-Drob (42), Carol E Franz (43), William S Kremen (43) (44), Michael J Lyons (45), Paul Lichtenstein (19), Meike Bartels (46), Catharina EM van Beijsterveldt (46), Gonneke Willemsen (46), Sevgi Y Öncel (47), Fazil Aliev (48), Hoe-Uk Jeong (49), Yoon-Mi Hur (49), Eric Turkheimer (50), Dorret I Boomsma (46), Thorkild IA Sørensen (51) (52), Jaakko Kaprio (2) (6), Karri Silventoinen (3) (53)

1. Department of Physiology, Faculty of Medicine and Nursing, University of the Basque Country, Bilbao 48080, Spain.

2. Department of Public Health, University of Helsinki, Helsinki 00014, Finland.

3. Department of Social Research, University of Helsinki, Helsinki 00014, Finland.

4. Institute of Clinical Medicine, University of Eastern Finland, Kuopio 70211, Finland.

5. Department of Public Health Nursing, Osaka City University, Osaka 545-0051, Japan.

6. Institute for Molecular Medicine FIMM, Helsinki 00014, Finland.

7. Department of Psychology, Ochanomizu University, Tokyo 112-8610, Japan.

8. Center for Forensic Mental Health, Chiba University, Chiba 260-8670, Japan.

9. Institute for Education and Human Development, Ochanomizu University, Tokyo 112-8610.

10. Department of Physical Education and Sport, University of Madeira, Funchal 9020-105, Portugal.

11. CIFI2D, Faculty of Sport, University of Porto, Porto 4200-450, Portugal.

12. The Hebrew University of Jerusalem, Jerusalem 91905, Israel.

13. Hadassah Hospital Obstetrics and Gynecology Department, Hebrew University Medical School, Jerusalem 91905, Israel.

14. Department of Noncommunicable Diseases Prevention, Qingdao Centers for Disease Control and Prevention, Qingdao 266033, China.

15. Department of Genetics, Physical Anthropology and Animal Physiology, University of the Basque Country UPV/EHU, Bilbao 48080, Spain.

16. Boston University, Department of Psychological and Brain Sciences, MA 02215, MA, USA.

17. Twins Research Australia, Centre for Epidemiology and Biostatistics, The University of Melbourne, Melbourne, Victoria 3010, Australia.

18. Department of Epidemiology, School of Public Health, Seoul National University, Seoul 08826, Korea.

19. Department of Medical Epidemiology and Biostatistics, Karolinska Institutet, Stockholm 17177, Sweden.

20. Pediatric Allergy and Pulmonology Unit at Astrid Lindgren Children’s Hospital, Karolinska University Hospital, Stockholm 17176, Sweden.

21. Department of Preventive Medicine, Keck School of Medicine of USC, University of Southern California, Los Angeles, California 90089, USA.

22. USC Norris Comprehensive Cancer Center, Los Angeles 90089, California, USA.

23. Department of Health and Exercise Sciences and Colorado School of Public Health, Colorado State University, Colorado 80523, USA.

24. Department of Psychology, Wayne State University, Detroit 48202, MI., USA.

25. Institute of Health and Environment, Seoul National University, Seoul 08826, South-Korea.

26. Health Behaviour Research Centre, Department of Epidemiology and Public Health, Institute of Epidemiology and Health Care, University College London, London WC1E 7HB, UK.

27. Istituto Superiore di Sanità - Centre for Behavioural Sciences and Mental Health, Rome 00161, Italy.

28. Department of Psychology, University of Southern California, Los Angeles, CA 90089, USA.

29. School of Law, Psychology and Social Work, Örebro University, Örebro 701 82, Sweden.

30. Institute for Behavioral Genetics, University of Colorado, Boulder, Colorado 80303, USA.

31. Institute of Behavioral Science, University of Colorado, Boulder, Colorado 80303, USA.

32. Centre of Human Genetics, University Hospitals Leuven, Leuven B-3000, Belgium.

33. Department of Obstetrics and Gynaecology, Ghent University Hospitals, Ghent 9820, Belgium.

34. The Charles Bronfman Institute for Personalized Medicine, The Mindich Child Health and Development Institute, Icahn School of Medicine at Mount Sinai, New York, NY 10029-5674, USA.

35. Michigan State University, East Lansing, Michigan 48823, USA.

36. Department of Human and Molecular Genetics, Virginia Institute for Psychiatric and Behavioral Genetics, Virginia Commonwealth University, Richmond, Virginia 23284, USA.

37. Department of Human and Molecular Genetics, Psychiatry & Massey Cancer Center, Virginia Commonwealth University, Richmond, Virginia 23284, USA.

38. Department of Psychology, University of Minnesota, Minneapolis, MN 55455, USA.

39. Center for Economic and Social Research, University of Southern California, Los Angeles, CA 90089, USA.

40. Health and Medicine Division, The National Academies of Sciences, Engineering, and Medicine, Washington, DC 20001, USA.

41. Norwegian Institute of Public Health, Oslo 0213, Norway.

42. Department of Psychology, University of Texas at Austin, Austin, TX 78712, USA.

43. Department of Psychiatry, University of California, San Diego, CA 92093, USA.

44. VA San Diego Center of Excellence for Stress and Mental Health, La Jolla, CA 92093, USA.

45. Boston University, Department of Psychology, Boston, MA 02215, USA.

46. Department of Biological Psychology, VU University Amsterdam, Amsterdam 1081, Netherlands.

47. Department of Statistics, Faculty of Arts and Sciences, Kırıkkale University, Kırıkkale 71450, Turkey.

48. Psychology and African American Studies, Virginia Commonwealth University, Richmond, Virginia 23284, USA.

49. Department of Education, Mokpo National University, Jeonnam 534-729, South Korea.

50. Department of Psychology, University of Virginia, Charlottesville, VA 22904, USA.

51. Novo Nordisk Foundation Centre for Basic Metabolic Research (Section of Metabolic Genetics), Faculty of Health and Medical Sciences, University of Copenhagen, Copenhagen 1353, Denmark.

52. Department of Public Health (Section of Epidemiology), Faculty of Health and Medical Sciences, University of Copenhagen, Copenhagen 1353, Denmark.

53. Osaka University Graduate School of Medicine, Osaka University, Osaka 565-0871, Japan.

**Supplementary files**

Supplementary table 1. Descriptive statistics of height (cm) and parental education (years) by age and cultural-geographic region.

|  | Males |  |  |  |  |  |  |  |  |  | Females | |  |  |  |  |  |  |  |  |
| --- | --- | --- | --- | --- | --- | --- | --- | --- | --- | --- | --- | --- | --- | --- | --- | --- | --- | --- | --- | --- |
|  |  | Height |  |  | Paternal education | |  | Maternal education | |  |  | | Height |  |  | Paternal education | |  | Maternal education | |
| Age | N | Mean | SD |  | Mean | SD |  | Mean | SD |  | N | | Mean | SD |  | Mean | SD |  | Mean | SD |
| Europe |  |  |  |  |  |  |  |  |  |  |  | |  |  |  |  |  |  |  |  |
| 1 | 12655 | 75.1 | 3.52 |  | 13.80 | 2.59 |  | 13.76 | 2.31 |  | 13095 | | 73.6 | 3.59 |  | 15.21 | 1.96 |  | 13.80 | 2.33 |
| 2 | 10233 | 87.7 | 3.88 |  | 13.86 | 2.70 |  | 13.88 | 2.40 |  | 10291 | | 86.4 | 3.97 |  | 15.37 | 1.82 |  | 13.91 | 2.43 |
| 3 | 9092 | 97.1 | 4.21 |  | 13.91 | 2.64 |  | 13.91 | 2.31 |  | 9568 | | 96.1 | 4.24 |  | 15.04 | 2.02 |  | 13.93 | 2.33 |
| 4 | 1628 | 102.7 | 4.85 |  | 13.37 | 3.74 |  | 14.28 | 3.47 |  | 1696 | | 101.5 | 4.63 |  | 15.14 | 1.93 |  | 14.43 | 3.44 |
| 5 | 5152 | 112.7 | 5.47 |  | 13.99 | 2.72 |  | 14.04 | 2.35 |  | 5288 | | 112.0 | 5.57 |  | 14.72 | 2.16 |  | 14.04 | 2.36 |
| 6 | 675 | 116.3 | 5.19 |  | 13.78 | 3.44 |  | 14.54 | 3.37 |  | 763 | | 115.0 | 5.03 |  | 15.17 | 1.82 |  | 14.70 | 3.33 |
| 7 | 5617 | 126.9 | 5.66 |  | 14.06 | 2.49 |  | 13.96 | 2.24 |  | 5979 | | 126.2 | 5.70 |  | 14.72 | 2.16 |  | 13.92 | 2.22 |
| 8 | 3193 | 130.3 | 5.78 |  | 13.90 | 2.79 |  | 14.00 | 2.62 |  | 3285 | | 129.5 | 5.68 |  | 15.11 | 2.03 |  | 13.95 | 2.59 |
| 9 | 1716 | 137.0 | 6.66 |  | 13.57 | 3.17 |  | 13.86 | 3.11 |  | 1688 | | 135.6 | 6.89 |  | 14.78 | 2.24 |  | 14.08 | 3.06 |
| 10 | 5509 | 143.2 | 6.67 |  | 14.05 | 2.55 |  | 13.90 | 2.33 |  | 5837 | | 142.5 | 6.80 |  | 14.38 | 2.65 |  | 13.79 | 2.23 |
| 11 | 2632 | 145.8 | 6.87 |  | 11.95 | 4.00 |  | 12.67 | 3.72 |  | 2616 | | 146.0 | 7.32 |  | 14.18 | 2.60 |  | 12.80 | 3.74 |
| 12 | 5343 | 153.7 | 7.68 |  | 13.54 | 3.10 |  | 13.51 | 2.70 |  | 5529 | | 154.8 | 7.78 |  | 14.70 | 2.49 |  | 13.50 | 2.70 |
| 13 | 1875 | 159.4 | 8.72 |  | 13.72 | 2.98 |  | 13.99 | 2.82 |  | 2039 | | 159.5 | 7.19 |  | 14.47 | 2.33 |  | 13.81 | 2.77 |
| 14 | 3789 | 166.1 | 9.02 |  | 12.17 | 4.06 |  | 12.76 | 3.71 |  | 4275 | | 163.1 | 6.66 |  | 14.47 | 2.45 |  | 12.92 | 3.62 |
| 15 | 1785 | 173.4 | 8.38 |  | 13.84 | 2.85 |  | 13.82 | 2.71 |  | 2035 | | 166.4 | 6.58 |  | 14.28 | 2.81 |  | 13.92 | 2.56 |
| 16 | 2382 | 176.2 | 7.52 |  | 12.21 | 3.03 |  | 12.20 | 2.92 |  | 2818 | | 165.9 | 6.26 |  | 14.90 | 2.10 |  | 12.33 | 2.94 |
| 17 | 3371 | 178.5 | 7.05 |  | 12.24 | 3.39 |  | 12.46 | 3.26 |  | 3851 | | 166.7 | 6.39 |  | 14.16 | 2.71 |  | 12.49 | 3.16 |
| 18 | 1845 | 179.6 | 7.11 |  | 11.46 | 3.88 |  | 11.95 | 3.44 |  | 2277 | | 167.2 | 6.52 |  | 14.49 | 2.46 |  | 11.90 | 3.35 |
| 19 | 1190 | 180.4 | 6.92 |  | 12.13 | 3.00 |  | 11.96 | 2.71 |  | 1638 | | 167.8 | 6.32 |  | 13.91 | 2.53 |  | 12.27 | 2.72 |
| 20-69 | 5752 | 178.9 | 6.46 |  | 10.66 | 3.10 |  | 10.58 | 2.80 |  | 7262 | | 165.7 | 5.91 |  | 13.08 | 4.39 |  | 10.58 | 2.79 |
| North America and Australia | | | | | | | | | | | | | | | | | | | | |
| 1 |  |  |  |  |  |  |  |  |  |  |  | |  |  |  |  |  |  |  |  |
| 2 | 314 | 88.1 | 3.18 |  | 15.71 | 2.08 |  | 16.25 | 1.74 |  | 270 | | 87.2 | 3.43 |  | 15.67 | 2.21 |  | 16.21 | 1.80 |
| 3 | 920 | 96.8 | 5.06 |  | 16.30 | 2.91 |  | 16.96 | 2.70 |  | 952 | | 95.7 | 5.32 |  | 16.30 | 2.92 |  | 16.97 | 2.84 |
| 4 | 1347 | 101.5 | 6.46 |  | 16.30 | 3.15 |  | 16.86 | 3.03 |  | 1287 | | 100.2 | 6.45 |  | 16.33 | 3.20 |  | 17.08 | 2.98 |
| 5 | 708 | 108.5 | 7.25 |  | 16.10 | 3.24 |  | 17.06 | 3.08 |  | 634 | | 107.1 | 7.83 |  | 16.10 | 3.34 |  | 16.80 | 2.90 |
| 6 | 567 | 114.5 | 9.15 |  | 16.27 | 3.27 |  | 16.63 | 3.01 |  | 543 | | 114.2 | 8.50 |  | 16.52 | 3.22 |  | 16.82 | 3.00 |
| 7 | 534 | 121.7 | 9.04 |  | 16.37 | 3.31 |  | 16.78 | 3.00 |  | 562 | | 120.3 | 7.67 |  | 16.38 | 3.39 |  | 16.77 | 3.13 |
| 8 | 511 | 129.8 | 7.59 |  | 16.17 | 3.02 |  | 17.04 | 2.99 |  | 497 | | 127.7 | 8.84 |  | 16.33 | 3.06 |  | 17.16 | 3.00 |
| 9 | 1118 | 133.8 | 7.49 |  | 15.58 | 3.25 |  | 16.06 | 3.11 |  | 1098 | | 133.5 | 7.70 |  | 15.70 | 3.40 |  | 16.08 | 3.23 |
| 10 | 699 | 139.3 | 7.40 |  | 15.70 | 3.35 |  | 16.11 | 3.16 |  | 661 | | 138.5 | 8.27 |  | 15.51 | 3.26 |  | 15.90 | 3.06 |
| 11 | 582 | 143.8 | 8.42 |  | 15.84 | 3.34 |  | 16.06 | 3.09 |  | 566 | | 144.9 | 8.65 |  | 15.58 | 3.43 |  | 15.87 | 3.22 |
| 12 | 673 | 150.9 | 9.21 |  | 15.45 | 3.10 |  | 15.57 | 2.86 |  | 707 | | 151.7 | 8.90 |  | 15.69 | 3.17 |  | 15.61 | 2.84 |
| 13 | 583 | 157.0 | 10.47 |  | 15.73 | 3.20 |  | 15.70 | 2.85 |  | 591 | | 156.1 | 7.85 |  | 15.65 | 3.07 |  | 15.59 | 2.74 |
| 14 | 712 | 165.9 | 9.24 |  | 15.29 | 2.99 |  | 15.39 | 2.77 |  | 746 | | 161.0 | 6.72 |  | 15.09 | 3.07 |  | 15.21 | 2.72 |
| 15 | 676 | 171.2 | 8.90 |  | 15.44 | 3.28 |  | 15.55 | 3.00 |  | 608 | | 162.5 | 7.25 |  | 15.37 | 3.29 |  | 15.45 | 2.98 |
| 16 | 812 | 175.1 | 8.14 |  | 15.55 | 3.03 |  | 15.35 | 2.77 |  | 852 | | 163.6 | 7.02 |  | 15.29 | 3.02 |  | 15.19 | 2.76 |
| 17 | 1043 | 176.4 | 7.88 |  | 14.36 | 3.78 |  | 14.46 | 3.36 |  | 993 | | 164.6 | 7.14 |  | 15.22 | 2.90 |  | 15.16 | 2.58 |
| 18 | 1347 | 175.0 | 7.35 |  | 11.77 | 4.37 |  | 12.22 | 3.90 |  | 635 | | 164.8 | 7.53 |  | 14.87 | 3.00 |  | 15.01 | 2.81 |
| 19 | 624 | 176.3 | 7.74 |  | 12.39 | 3.88 |  | 12.57 | 3.42 |  | 602 | | 164.1 | 6.98 |  | 14.49 | 2.93 |  | 14.32 | 2.72 |
| 20-69 | 19181 | 178.5 | 7.21 |  | 12.15 | 3.68 |  | 12.34 | 3.08 |  | 22419 | | 164.5 | 6.86 |  | 12.78 | 3.44 |  | 12.77 | 2.96 |
| East Asia | | | | | | | | | | | | | | | | | | | | |
| 1 | 500 | 74.0 | 5.10 |  | 15.21 | 1.96 |  | 14.56 | 1.71 |  | | 536 | 72.8 | 5.28 |  | 15.30 | 1.86 |  | 14.44 | 1.71 |
| 2 | 365 | 81.2 | 3.49 |  | 15.37 | 1.82 |  | 14.70 | 1.69 |  | | 369 | 79.7 | 4.17 |  | 15.39 | 1.78 |  | 14.65 | 1.69 |
| 3 | 529 | 92.1 | 4.20 |  | 15.04 | 2.02 |  | 14.39 | 1.71 |  | | 567 | 90.7 | 4.15 |  | 14.97 | 1.98 |  | 14.17 | 1.74 |
| 4 | 326 | 97.8 | 4.92 |  | 15.14 | 1.93 |  | 14.29 | 1.68 |  | | 326 | 96.8 | 4.47 |  | 15.29 | 1.91 |  | 14.36 | 1.70 |
| 5 | 390 | 104.7 | 5.26 |  | 14.72 | 2.16 |  | 14.00 | 1.81 |  | | 404 | 103.6 | 4.66 |  | 14.83 | 2.11 |  | 13.88 | 1.75 |
| 6 | 437 | 111.1 | 5.57 |  | 15.17 | 1.82 |  | 14.13 | 1.58 |  | | 443 | 110.6 | 5.08 |  | 15.31 | 1.76 |  | 14.10 | 1.67 |
| 7 | 470 | 117.6 | 5.36 |  | 14.72 | 2.16 |  | 14.19 | 1.80 |  | | 448 | 116.7 | 5.61 |  | 14.97 | 2.03 |  | 14.19 | 1.72 |
| 8 | 417 | 123.8 | 5.72 |  | 15.11 | 2.03 |  | 14.14 | 1.77 |  | | 463 | 122.5 | 5.84 |  | 15.26 | 1.90 |  | 14.11 | 1.78 |
| 9 | 457 | 129.2 | 5.70 |  | 14.78 | 2.24 |  | 14.14 | 1.95 |  | | 459 | 128.5 | 6.28 |  | 14.93 | 2.08 |  | 14.03 | 1.78 |
| 10 | 505 | 135.9 | 6.68 |  | 14.38 | 2.65 |  | 13.56 | 2.57 |  | | 561 | 135.7 | 7.22 |  | 14.90 | 2.39 |  | 13.77 | 2.35 |
| 11 | 535 | 141.6 | 6.84 |  | 14.18 | 2.60 |  | 13.51 | 2.35 |  | | 597 | 142.3 | 7.83 |  | 14.33 | 2.64 |  | 13.39 | 2.65 |
| 12 | 506 | 147.6 | 7.89 |  | 14.70 | 2.49 |  | 13.81 | 2.16 |  | | 514 | 148.5 | 7.62 |  | 14.71 | 2.55 |  | 13.68 | 2.40 |
| 13 | 376 | 155.8 | 8.70 |  | 14.47 | 2.33 |  | 13.91 | 2.18 |  | | 472 | 152.6 | 6.56 |  | 14.59 | 2.48 |  | 13.67 | 2.25 |
| 14 | 359 | 162.6 | 7.42 |  | 14.47 | 2.45 |  | 13.55 | 2.45 |  | | 381 | 155.2 | 5.99 |  | 14.90 | 2.29 |  | 13.72 | 2.40 |
| 15 | 281 | 167.4 | 6.68 |  | 14.28 | 2.81 |  | 13.65 | 2.47 |  | | 373 | 156.6 | 5.70 |  | 14.47 | 2.71 |  | 13.70 | 2.19 |
| 16 | 276 | 168.5 | 5.89 |  | 14.90 | 2.10 |  | 13.79 | 2.52 |  | | 302 | 157.0 | 5.60 |  | 14.84 | 2.17 |  | 13.82 | 2.26 |
| 17 | 249 | 170.7 | 6.04 |  | 14.16 | 2.71 |  | 13.41 | 2.42 |  | | 319 | 157.5 | 5.64 |  | 14.64 | 2.18 |  | 13.84 | 1.86 |
| 18 | 269 | 170.4 | 5.82 |  | 14.49 | 2.46 |  | 13.49 | 2.09 |  | | 285 | 157.4 | 5.55 |  | 14.65 | 2.49 |  | 13.60 | 2.18 |
| 19 | 248 | 172.8 | 5.98 |  | 13.91 | 2.53 |  | 13.48 | 2.16 |  | | 292 | 157.7 | 5.52 |  | 14.46 | 2.43 |  | 13.72 | 2.12 |
| 20-69 | 790 | 170.8 | 6.06 |  | 13.08 | 4.39 |  | 11.77 | 4.47 |  | | 1350 | 158.1 | 5.70 |  | 12.61 | 4.44 |  | 10.99 | 4.58 |

Supplementary table 2. Additive genetic, shared environmental and unique environmental variances of height with 95% confidence intervals by age, sex and parental education for all cohorts together.

|  | Additive genetic  variance | | | Shared environmental  variance | | | Unique environmental  variance | | |  | Additive genetic  variance | | | Shared environmental  variance | | | Unique environmental  variance | | |
| --- | --- | --- | --- | --- | --- | --- | --- | --- | --- | --- | --- | --- | --- | --- | --- | --- | --- | --- | --- |
| Age | a^2^ | LL | UL | c^2^ | LL | UL | e^2^ | LL | UL |  | a^2^ | LL | UL | c^2^ | LL | UL | e^2^ | LL | UL |
| Males with low parental education | | | | | | | | | |  | Females with low parental education | | | | | | | | |
| 1 | 3.58 | 3.01 | 4.22 | 3.51 | 2.83 | 4.17 | 0.96 | 0.86 | 1.07 |  | 4.14 | 3.49 | 4.86 | 2.84 | 2.09 | 3.54 | 1.06 | 0.96 | 1.17 |
| 2 | 7.42 | 6.34 | 8.63 | 3.09 | 2.25 | 4.24 | 1.26 | 1.12 | 1.42 |  | 6.95 | 5.89 | 8.16 | 3.70 | 3.61 | 4.87 | 1.23 | 1.10 | 1.38 |
| 3 | 10.46 | 8.92 | 12.24 | 4.06 | 3.20 | 5.76 | 1.56 | 1.38 | 1.76 |  | 11.06 | 9.44 | 12.88 | 3.73 | 2.36 | 5.43 | 1.93 | 1.73 | 2.17 |
| 4 | 9.98 | 7.20 | 13.36 | 17.39 | 13.45 | 21.20 | 2.68 | 2.23 | 3.27 |  | 12.66 | 9.11 | 17.08 | 11.16 | 6.53 | 15.30 | 3.09 | 2.59 | 3.74 |
| 5 | 22.35 | 18.66 | 26.80 | 6.99 | 1.35 | 11.13 | 1.88 | 1.61 | 2.21 |  | 19.64 | 16.07 | 24.00 | 12.27 | 8.78 | 16.54 | 2.28 | 1.97 | 2.67 |
| 6 | 14.67 | 8.82 | 22.57 | 29.61 | 20.45 | 38.40 | 4.09 | 3.20 | 5.34 |  | 30.57 | 22.45 | 38.02 | 3.46 | 0.00 | 12.10 | 2.49 | 1.93 | 3.29 |
| 7 | 22.91 | 19.20 | 27.35 | 11.09 | 6.43 | 15.42 | 2.15 | 1.85 | 2.53 |  | 20.78 | 17.21 | 25.02 | 12.08 | 7.60 | 16.27 | 2.64 | 2.28 | 3.07 |
| 8 | 19.32 | 15.27 | 24.42 | 18.35 | 12.71 | 23.71 | 2.14 | 1.78 | 2.62 |  | 24.42 | 19.24 | 31.03 | 9.95 | 1.00 | 15.88 | 2.23 | 1.88 | 2.68 |
| 9 | 22.92 | 17.02 | 30.50 | 21.11 | 15.81 | 28.49 | 4.05 | 3.39 | 4.89 |  | 21.55 | 16.23 | 28.50 | 23.76 | 15.99 | 30.93 | 2.89 | 2.39 | 3.55 |
| 10 | 39.72 | 33.46 | 46.56 | 4.77 | 0.00 | 11.49 | 2.52 | 2.16 | 2.95 |  | 33.72 | 27.84 | 40.80 | 9.38 | 5.12 | 15.82 | 3.67 | 3.19 | 4.24 |
| 11 | 31.19 | 24.45 | 39.46 | 16.58 | 8.05 | 24.30 | 5.12 | 4.35 | 6.08 |  | 47.06 | 38.26 | 56.58 | 6.22 | 0.00 | 15.55 | 4.15 | 3.54 | 4.91 |
| 12 | 42.90 | 35.22 | 52.22 | 9.12 | 0.00 | 17.46 | 4.64 | 4.03 | 5.38 |  | 44.09 | 36.25 | 52.19 | 5.45 | 0.00 | 13.76 | 5.44 | 4.74 | 6.27 |
| 13 | 68.67 | 49.18 | 87.08 | 12.20 | 0.00 | 33.82 | 7.53 | 6.19 | 9.28 |  | 35.06 | 26.26 | 44.49 | 10.63 | 4.37 | 20.43 | 4.90 | 4.07 | 5.96 |
| 14 | 67.42 | 55.49 | 76.44 | 3.66 | 0.00 | 16.12 | 6.56 | 5.66 | 7.66 |  | 29.89 | 23.85 | 36.61 | 5.06 | 0.00 | 11.34 | 5.35 | 4.66 | 6.19 |
| 15 | 55.40 | 41.94 | 69.93 | 8.41 | 0.00 | 23.04 | 5.85 | 4.71 | 7.38 |  | 33.22 | 25.00 | 40.75 | 3.47 | 0.00 | 12.19 | 3.95 | 3.24 | 4.88 |
| 16 | 26.72 | 18.67 | 37.04 | 21.96 | 16.34 | 31.49 | 6.25 | 5.18 | 7.64 |  | 25.16 | 19.10 | 32.74 | 11.99 | 7.20 | 18.88 | 4.04 | 3.38 | 4.88 |
| 17 | 36.09 | 28.23 | 45.22 | 6.95 | 0.00 | 15.42 | 5.53 | 4.72 | 6.53 |  | 22.63 | 17.07 | 29.47 | 11.24 | 9.36 | 17.39 | 5.08 | 4.38 | 5.93 |
| 18 | 37.44 | 30.26 | 46.37 | 8.88 | 0.00 | 16.96 | 3.19 | 2.69 | 3.81 |  | 27.65 | 20.94 | 36.20 | 8.43 | 0.00 | 15.83 | 4.12 | 3.45 | 4.97 |
| 19 | 30.49 | 21.81 | 42.06 | 10.74 | 0.00 | 20.64 | 4.28 | 3.43 | 5.42 |  | 29.69 | 21.86 | 36.20 | 2.47 | 0.00 | 10.88 | 4.05 | 3.34 | 4.98 |
| 20-69 | 34.08 | 30.99 | 37.42 | 7.92 | 4.62 | 11.12 | 5.93 | 5.55 | 6.34 |  | 29.44 | 26.94 | 32.13 | 8.70 | 7.40 | 11.33 | 5.59 | 5.30 | 5.91 |
| Males with intermediate parental education | | | | | | | | | |  | Females with intermediate parental education | | | | | | | | |
| 1 | 3.44 | 2.94 | 3.99 | 2.96 | 2.40 | 3.50 | 1.08 | 0.99 | 1.18 |  | 2.98 | 2.48 | 3.52 | 3.22 | 2.66 | 3.75 | 1.23 | 1.13 | 1.33 |
| 2 | 6.77 | 5.94 | 7.69 | 2.48 | 2.06 | 3.37 | 1.17 | 1.06 | 1.29 |  | 7.83 | 6.89 | 8.89 | 1.78 | 0.03 | 2.77 | 1.11 | 1.01 | 1.22 |
| 3 | 10.49 | 9.30 | 11.82 | 3.58 | 3.41 | 4.88 | 1.26 | 1.14 | 1.40 |  | 9.36 | 8.22 | 10.62 | 4.57 | 3.51 | 5.82 | 1.42 | 1.29 | 1.56 |
| 4 | 13.06 | 10.23 | 16.68 | 13.04 | 11.12 | 16.96 | 1.28 | 1.03 | 1.63 |  | 14.72 | 11.30 | 19.14 | 8.41 | 0.39 | 12.49 | 1.60 | 1.31 | 1.99 |
| 5 | 17.16 | 14.82 | 19.89 | 8.28 | 5.36 | 11.00 | 1.39 | 1.21 | 1.59 |  | 16.72 | 14.15 | 19.76 | 8.01 | 4.82 | 10.94 | 1.77 | 1.56 | 2.01 |
| 6 | 18.02 | 12.97 | 25.10 | 17.99 | 9.98 | 25.47 | 1.81 | 1.38 | 2.45 |  | 18.91 | 14.16 | 25.35 | 15.94 | 10.01 | 22.45 | 1.64 | 1.30 | 2.11 |
| 7 | 20.41 | 17.76 | 23.47 | 9.46 | 8.97 | 12.52 | 1.76 | 1.57 | 1.99 |  | 19.45 | 16.71 | 22.60 | 8.60 | 5.39 | 11.65 | 2.51 | 2.25 | 2.80 |
| 8 | 22.80 | 18.88 | 27.61 | 7.47 | 5.20 | 11.94 | 1.85 | 1.57 | 2.19 |  | 24.03 | 19.78 | 29.16 | 6.66 | 2.96 | 11.30 | 2.50 | 2.14 | 2.93 |
| 9 | 25.20 | 19.29 | 32.50 | 10.85 | 5.27 | 17.40 | 4.00 | 3.29 | 4.92 |  | 36.69 | 28.47 | 46.08 | 6.58 | 0.00 | 15.65 | 2.71 | 2.27 | 3.26 |
| 10 | 32.27 | 27.91 | 37.35 | 6.53 | 2.10 | 11.25 | 2.54 | 2.24 | 2.88 |  | 28.01 | 23.96 | 32.71 | 13.45 | 8.54 | 18.00 | 3.47 | 3.11 | 3.88 |
| 11 | 33.39 | 26.43 | 42.11 | 10.07 | 2.55 | 17.96 | 3.59 | 2.96 | 4.43 |  | 42.98 | 34.18 | 54.35 | 12.44 | 2.96 | 22.51 | 2.55 | 2.14 | 3.07 |
| 12 | 46.99 | 40.54 | 53.81 | 4.76 | 0.00 | 11.52 | 3.78 | 3.36 | 4.28 |  | 44.06 | 37.11 | 52.13 | 6.97 | 0.00 | 14.29 | 5.88 | 5.24 | 6.64 |
| 13 | 38.44 | 28.50 | 50.78 | 29.23 | 16.69 | 41.01 | 8.00 | 6.52 | 9.96 |  | 44.41 | 35.34 | 51.30 | 2.37 | 0.00 | 11.93 | 3.55 | 2.99 | 4.25 |
| 14 | 62.46 | 49.37 | 74.95 | 7.40 | 0.00 | 21.39 | 7.17 | 6.07 | 8.57 |  | 29.29 | 23.65 | 35.92 | 5.39 | 0.00 | 11.35 | 4.33 | 3.74 | 5.05 |
| 15 | 51.12 | 38.86 | 63.40 | 7.35 | 0.00 | 20.23 | 8.05 | 6.61 | 9.93 |  | 28.85 | 22.14 | 36.73 | 5.96 | 0.00 | 13.23 | 3.92 | 3.32 | 4.66 |
| 16 | 43.08 | 34.93 | 52.70 | 7.64 | 0.00 | 16.32 | 5.15 | 4.37 | 6.13 |  | 33.53 | 28.55 | 36.30 | 0.00 | 0.00 | 5.02 | 3.44 | 2.99 | 3.98 |
| 17 | 37.96 | 31.19 | 45.29 | 5.11 | 0.00 | 12.14 | 5.15 | 4.45 | 6.02 |  | 26.93 | 22.23 | 32.49 | 6.30 | 1.82 | 11.29 | 3.98 | 3.52 | 4.52 |
| 18 | 41.77 | 35.71 | 46.62 | 0.00 | 0.00 | 5.46 | 6.26 | 5.19 | 7.65 |  | 27.22 | 20.48 | 33.84 | 3.85 | 0.00 | 10.79 | 5.27 | 4.45 | 6.31 |
| 19 | 34.78 | 25.66 | 43.59 | 4.71 | 0.00 | 14.44 | 4.55 | 3.71 | 5.66 |  | 25.18 | 18.28 | 32.78 | 5.15 | 0.00 | 12.33 | 5.53 | 4.63 | 6.69 |
| 20-69 | 30.88 | 28.28 | 33.67 | 9.74 | 9.01 | 12.50 | 5.05 | 4.75 | 5.38 |  | 28.46 | 26.21 | 30.88 | 6.55 | 5.78 | 8.88 | 5.01 | 4.75 | 5.29 |
| Males with high parental education | | | | | | | | | |  | Females with high parental education | | | | | | | | |
| 1 | 4.01 | 3.27 | 4.83 | 2.38 | 1.55 | 3.16 | 1.10 | 0.98 | 1.25 |  | 3.88 | 3.16 | 4.69 | 2.51 | 1.70 | 3.26 | 1.19 | 1.07 | 1.34 |
| 2 | 7.09 | 6.00 | 8.35 | 2.17 | 1.35 | 3.33 | 1.16 | 1.02 | 1.32 |  | 5.90 | 4.94 | 6.73 | 3.35 | 0.67 | 4.41 | 1.01 | 0.89 | 1.14 |
| 3 | 9.34 | 7.91 | 10.98 | 4.35 | 3.34 | 5.92 | 1.37 | 1.20 | 1.57 |  | 9.47 | 8.02 | 11.15 | 3.71 | 2.26 | 5.29 | 1.36 | 1.20 | 1.54 |
| 4 | 15.85 | 12.30 | 20.26 | 5.84 | 3.48 | 9.79 | 2.11 | 1.72 | 2.63 |  | 13.04 | 10.34 | 16.45 | 8.97 | 7.62 | 12.37 | 1.30 | 1.07 | 1.61 |
| 5 | 18.15 | 15.24 | 21.65 | 6.44 | 3.82 | 9.78 | 1.36 | 1.16 | 1.60 |  | 17.62 | 14.53 | 21.33 | 7.12 | 3.33 | 10.58 | 1.98 | 1.71 | 2.30 |
| 6 | 20.39 | 13.73 | 29.22 | 19.78 | 10.53 | 28.43 | 3.65 | 2.76 | 4.95 |  | 26.62 | 19.61 | 36.32 | 9.30 | 0.00 | 17.89 | 2.13 | 1.62 | 2.88 |
| 7 | 19.44 | 16.34 | 23.16 | 12.45 | 12.12 | 16.26 | 1.76 | 1.51 | 2.08 |  | 23.65 | 19.76 | 28.32 | 5.18 | 0.50 | 9.40 | 2.16 | 1.89 | 2.50 |
| 8 | 25.23 | 19.99 | 31.88 | 7.74 | 2.07 | 13.67 | 2.41 | 2.01 | 2.93 |  | 25.12 | 20.07 | 31.55 | 9.14 | 5.24 | 14.97 | 1.99 | 1.67 | 2.40 |
| 9 | 33.60 | 26.84 | 40.85 | 4.49 | 0.00 | 11.87 | 2.09 | 1.73 | 2.56 |  | 26.64 | 20.70 | 34.15 | 9.54 | 5.36 | 16.22 | 3.08 | 2.56 | 3.74 |
| 10 | 30.00 | 25.45 | 35.40 | 10.36 | 4.82 | 15.50 | 2.37 | 2.05 | 2.76 |  | 26.74 | 22.03 | 32.49 | 14.70 | 13.03 | 20.22 | 2.58 | 2.25 | 2.99 |
| 11 | 27.36 | 19.75 | 36.72 | 11.80 | 4.43 | 19.94 | 6.53 | 5.39 | 8.02 |  | 46.64 | 39.58 | 51.33 | 0.00 | 0.00 | 7.01 | 3.56 | 2.97 | 4.31 |
| 12 | 38.04 | 31.57 | 45.71 | 9.78 | 9.73 | 16.75 | 4.60 | 3.98 | 5.34 |  | 44.86 | 37.81 | 53.22 | 7.02 | 0.00 | 14.58 | 3.59 | 3.13 | 4.14 |
| 13 | 44.30 | 33.71 | 57.84 | 20.30 | 16.90 | 32.61 | 5.52 | 4.48 | 6.93 |  | 35.33 | 26.83 | 45.84 | 7.90 | 0.00 | 17.20 | 3.69 | 3.04 | 4.53 |
| 14 | 53.06 | 42.85 | 63.50 | 6.78 | 0.00 | 17.60 | 6.43 | 5.50 | 7.56 |  | 26.53 | 21.03 | 33.11 | 8.12 | 2.97 | 13.99 | 4.79 | 4.14 | 5.58 |
| 15 | 36.87 | 26.04 | 51.42 | 22.93 | 20.44 | 35.72 | 6.17 | 5.00 | 7.73 |  | 33.87 | 25.33 | 40.90 | 3.07 | 0.00 | 12.25 | 3.93 | 3.22 | 4.87 |
| 16 | 40.53 | 31.27 | 49.57 | 4.63 | 0.00 | 14.40 | 4.93 | 4.04 | 6.11 |  | 23.55 | 17.44 | 31.20 | 6.60 | 0.00 | 13.19 | 4.35 | 3.59 | 5.33 |
| 17 | 38.64 | 31.27 | 47.46 | 7.02 | 0.00 | 15.11 | 4.05 | 3.43 | 4.82 |  | 23.00 | 17.22 | 29.99 | 9.17 | 4.44 | 15.34 | 5.64 | 4.83 | 6.62 |
| 18 | 29.03 | 22.73 | 36.85 | 8.31 | 1.37 | 15.26 | 3.95 | 3.32 | 4.74 |  | 30.08 | 22.67 | 39.16 | 7.11 | 0.00 | 15.08 | 4.84 | 3.97 | 5.99 |
| 19 | 34.13 | 22.64 | 44.04 | 4.10 | 0.00 | 16.18 | 6.09 | 4.76 | 7.99 |  | 25.41 | 18.42 | 32.64 | 3.91 | 0.00 | 11.31 | 4.04 | 3.28 | 5.04 |
| 20-69 | 24.67 | 21.75 | 27.84 | 15.44 | 12.20 | 18.56 | 7.33 | 6.86 | 7.84 |  | 26.76 | 24.11 | 29.63 | 11.22 | 9.48 | 14.03 | 6.13 | 5.77 | 6.51 |

LL: lower limit of confidence interval; UL: upper limit

Supplementary table 3. Proportion of the height variance explained by additive genetic, shared environmental and unique environmental with 95% confidence intervals by age, sex and parental education for all cohorts together.

|  | Additive genetic  variance | | | Shared environmental  variance | | | Unique environmental  variance | | |  | Additive genetic  variance | | | Shared environmental  variance | | | Unique environmental  variance | | |
| --- | --- | --- | --- | --- | --- | --- | --- | --- | --- | --- | --- | --- | --- | --- | --- | --- | --- | --- | --- |
| Age | a^2^ | LL | UL | c^2^ | LL | UL | e^2^ | LL | UL |  | a^2^ | LL | UL | c^2^ | LL | UL | e^2^ | LL | UL |
| Males with low parental education | | | | | | | | | |  | Females with low parental education | | | | | | | | |
| 1 | 0.44 | 0.37 | 0.53 | 0.44 | 0.36 | 0.51 | 0.12 | 0.11 | 0.13 |  | 0.51 | 0.43 | 0.61 | 0.35 | 0.26 | 0.43 | 0.13 | 0.12 | 0.15 |
| 2 | 0.63 | 0.54 | 0.73 | 0.26 | 0.16 | 0.35 | 0.11 | 0.09 | 0.12 |  | 0.59 | 0.49 | 0.69 | 0.31 | 0.21 | 0.40 | 0.10 | 0.09 | 0.12 |
| 3 | 0.65 | 0.55 | 0.76 | 0.25 | 0.14 | 0.35 | 0.10 | 0.09 | 0.11 |  | 0.66 | 0.56 | 0.77 | 0.22 | 0.11 | 0.32 | 0.12 | 0.10 | 0.13 |
| 4 | 0.33 | 0.24 | 0.45 | 0.58 | 0.46 | 0.67 | 0.09 | 0.07 | 0.11 |  | 0.47 | 0.34 | 0.64 | 0.41 | 0.25 | 0.54 | 0.11 | 0.09 | 0.14 |
| 5 | 0.72 | 0.59 | 0.86 | 0.22 | 0.08 | 0.35 | 0.06 | 0.05 | 0.07 |  | 0.57 | 0.47 | 0.71 | 0.36 | 0.23 | 0.47 | 0.07 | 0.06 | 0.08 |
| 6 | 0.30 | 0.18 | 0.47 | 0.61 | 0.45 | 0.73 | 0.08 | 0.06 | 0.11 |  | 0.84 | 0.62 | 0.94 | 0.09 | 0.00 | 0.32 | 0.07 | 0.05 | 0.09 |
| 7 | 0.63 | 0.53 | 0.76 | 0.31 | 0.18 | 0.41 | 0.06 | 0.05 | 0.07 |  | 0.59 | 0.48 | 0.71 | 0.34 | 0.22 | 0.44 | 0.07 | 0.06 | 0.09 |
| 8 | 0.49 | 0.38 | 0.62 | 0.46 | 0.33 | 0.57 | 0.05 | 0.04 | 0.07 |  | 0.67 | 0.52 | 0.85 | 0.27 | 0.09 | 0.42 | 0.06 | 0.05 | 0.07 |
| 9 | 0.48 | 0.35 | 0.64 | 0.44 | 0.28 | 0.56 | 0.08 | 0.07 | 0.10 |  | 0.45 | 0.33 | 0.60 | 0.49 | 0.34 | 0.61 | 0.06 | 0.05 | 0.08 |
| 10 | 0.85 | 0.71 | 0.95 | 0.10 | 0.00 | 0.24 | 0.05 | 0.05 | 0.06 |  | 0.72 | 0.59 | 0.87 | 0.20 | 0.05 | 0.33 | 0.08 | 0.07 | 0.09 |
| 11 | 0.59 | 0.46 | 0.75 | 0.31 | 0.16 | 0.44 | 0.10 | 0.08 | 0.12 |  | 0.82 | 0.66 | 0.94 | 0.11 | 0.00 | 0.26 | 0.07 | 0.06 | 0.09 |
| 12 | 0.76 | 0.62 | 0.92 | 0.16 | 0.00 | 0.30 | 0.08 | 0.07 | 0.10 |  | 0.80 | 0.66 | 0.91 | 0.10 | 0.00 | 0.24 | 0.10 | 0.08 | 0.12 |
| 13 | 0.75 | 0.55 | 0.93 | 0.17 | 0.00 | 0.36 | 0.09 | 0.07 | 0.11 |  | 0.68 | 0.52 | 0.87 | 0.23 | 0.03 | 0.38 | 0.10 | 0.08 | 0.12 |
| 14 | 0.87 | 0.71 | 0.93 | 0.05 | 0.00 | 0.20 | 0.08 | 0.07 | 0.10 |  | 0.74 | 0.59 | 0.88 | 0.13 | 0.00 | 0.27 | 0.13 | 0.11 | 0.16 |
| 15 | 0.80 | 0.60 | 0.93 | 0.12 | 0.00 | 0.32 | 0.08 | 0.07 | 0.11 |  | 0.82 | 0.61 | 0.92 | 0.09 | 0.00 | 0.29 | 0.10 | 0.08 | 0.12 |
| 16 | 0.49 | 0.34 | 0.68 | 0.40 | 0.21 | 0.54 | 0.11 | 0.09 | 0.14 |  | 0.61 | 0.46 | 0.79 | 0.29 | 0.11 | 0.44 | 0.10 | 0.08 | 0.12 |
| 17 | 0.74 | 0.61 | 0.90 | 0.14 | 0.00 | 0.27 | 0.11 | 0.10 | 0.14 |  | 0.58 | 0.45 | 0.76 | 0.29 | 0.11 | 0.42 | 0.13 | 0.11 | 0.15 |
| 18 | 0.76 | 0.61 | 0.94 | 0.18 | 0.00 | 0.33 | 0.06 | 0.05 | 0.08 |  | 0.69 | 0.52 | 0.90 | 0.21 | 0.00 | 0.38 | 0.10 | 0.08 | 0.13 |
| 19 | 0.67 | 0.48 | 0.91 | 0.24 | 0.00 | 0.43 | 0.09 | 0.07 | 0.12 |  | 0.82 | 0.60 | 0.91 | 0.07 | 0.00 | 0.28 | 0.11 | 0.09 | 0.14 |
| 20-69 | 0.71 | 0.65 | 0.78 | 0.17 | 0.10 | 0.23 | 0.12 | 0.11 | 0.13 |  | 0.67 | 0.62 | 0.73 | 0.20 | 0.14 | 0.26 | 0.13 | 0.12 | 0.14 |
| Males with intermediate parental education | | | | | | | | | |  | Females with intermediate parental education | | | | | | | | |
| 1 | 0.46 | 0.39 | 0.53 | 0.40 | 0.32 | 0.46 | 0.14 | 0.13 | 0.16 |  | 0.40 | 0.33 | 0.47 | 0.43 | 0.36 | 0.50 | 0.17 | 0.15 | 0.18 |
| 2 | 0.65 | 0.57 | 0.72 | 0.24 | 0.15 | 0.32 | 0.11 | 0.10 | 0.12 |  | 0.73 | 0.64 | 0.83 | 0.17 | 0.13 | 0.25 | 0.10 | 0.09 | 0.11 |
| 3 | 0.68 | 0.60 | 0.77 | 0.23 | 0.15 | 0.31 | 0.08 | 0.07 | 0.09 |  | 0.61 | 0.53 | 0.69 | 0.30 | 0.22 | 0.37 | 0.09 | 0.08 | 0.10 |
| 4 | 0.48 | 0.37 | 0.61 | 0.48 | 0.34 | 0.58 | 0.05 | 0.04 | 0.06 |  | 0.60 | 0.45 | 0.78 | 0.34 | 0.16 | 0.48 | 0.06 | 0.05 | 0.08 |
| 5 | 0.64 | 0.55 | 0.74 | 0.31 | 0.20 | 0.40 | 0.05 | 0.04 | 0.06 |  | 0.63 | 0.53 | 0.75 | 0.30 | 0.19 | 0.40 | 0.07 | 0.06 | 0.08 |
| 6 | 0.48 | 0.34 | 0.67 | 0.48 | 0.28 | 0.62 | 0.05 | 0.04 | 0.07 |  | 0.52 | 0.38 | 0.70 | 0.44 | 0.26 | 0.57 | 0.04 | 0.03 | 0.06 |
| 7 | 0.65 | 0.56 | 0.74 | 0.30 | 0.20 | 0.39 | 0.06 | 0.05 | 0.06 |  | 0.64 | 0.54 | 0.74 | 0.28 | 0.18 | 0.37 | 0.08 | 0.07 | 0.09 |
| 8 | 0.71 | 0.58 | 0.86 | 0.23 | 0.08 | 0.36 | 0.06 | 0.05 | 0.07 |  | 0.72 | 0.59 | 0.88 | 0.20 | 0.05 | 0.33 | 0.08 | 0.06 | 0.09 |
| 9 | 0.63 | 0.48 | 0.81 | 0.27 | 0.09 | 0.42 | 0.10 | 0.08 | 0.12 |  | 0.80 | 0.61 | 0.95 | 0.14 | 0.00 | 0.33 | 0.06 | 0.05 | 0.07 |
| 10 | 0.78 | 0.67 | 0.90 | 0.16 | 0.03 | 0.27 | 0.06 | 0.05 | 0.07 |  | 0.62 | 0.53 | 0.73 | 0.30 | 0.19 | 0.39 | 0.08 | 0.07 | 0.09 |
| 11 | 0.71 | 0.56 | 0.90 | 0.21 | 0.03 | 0.37 | 0.08 | 0.06 | 0.10 |  | 0.74 | 0.58 | 0.94 | 0.21 | 0.06 | 0.37 | 0.04 | 0.04 | 0.05 |
| 12 | 0.85 | 0.73 | 0.94 | 0.09 | 0.00 | 0.20 | 0.07 | 0.06 | 0.08 |  | 0.77 | 0.65 | 0.90 | 0.12 | 0.00 | 0.25 | 0.10 | 0.09 | 0.12 |
| 13 | 0.51 | 0.37 | 0.67 | 0.39 | 0.22 | 0.52 | 0.11 | 0.08 | 0.13 |  | 0.88 | 0.70 | 0.94 | 0.05 | 0.00 | 0.23 | 0.07 | 0.06 | 0.09 |
| 14 | 0.81 | 0.64 | 0.92 | 0.10 | 0.00 | 0.27 | 0.09 | 0.08 | 0.11 |  | 0.75 | 0.60 | 0.90 | 0.14 | 0.00 | 0.28 | 0.11 | 0.09 | 0.13 |
| 15 | 0.77 | 0.58 | 0.90 | 0.11 | 0.00 | 0.29 | 0.12 | 0.10 | 0.15 |  | 0.74 | 0.57 | 0.91 | 0.15 | 0.00 | 0.33 | 0.10 | 0.08 | 0.12 |
| 16 | 0.77 | 0.62 | 0.92 | 0.14 | 0.00 | 0.28 | 0.09 | 0.08 | 0.11 |  | 0.91 | 0.77 | 0.92 | 0.00 | 0.00 | 0.13 | 0.09 | 0.08 | 0.11 |
| 17 | 0.79 | 0.65 | 0.91 | 0.11 | 0.00 | 0.25 | 0.11 | 0.09 | 0.13 |  | 0.72 | 0.60 | 0.87 | 0.17 | 0.02 | 0.30 | 0.11 | 0.09 | 0.12 |
| 18 | 0.87 | 0.76 | 0.89 | 0.00 | 0.00 | 0.11 | 0.13 | 0.11 | 0.16 |  | 0.75 | 0.56 | 0.88 | 0.11 | 0.00 | 0.29 | 0.15 | 0.12 | 0.18 |
| 19 | 0.79 | 0.58 | 0.92 | 0.11 | 0.00 | 0.32 | 0.10 | 0.08 | 0.13 |  | 0.70 | 0.51 | 0.87 | 0.14 | 0.00 | 0.33 | 0.15 | 0.13 | 0.19 |
| 20-69 | 0.68 | 0.62 | 0.74 | 0.21 | 0.15 | 0.27 | 0.11 | 0.10 | 0.12 |  | 0.71 | 0.65 | 0.77 | 0.16 | 0.10 | 0.22 | 0.13 | 0.12 | 0.13 |
| Males with high parental education | | | | | | | | | |  | Females with high parental education | | | | | | | | |
| 1 | 0.53 | 0.44 | 0.62 | 0.32 | 0.21 | 0.41 | 0.15 | 0.13 | 0.17 |  | 0.51 | 0.42 | 0.62 | 0.33 | 0.23 | 0.42 | 0.16 | 0.14 | 0.18 |
| 2 | 0.68 | 0.57 | 0.80 | 0.21 | 0.10 | 0.31 | 0.11 | 0.10 | 0.13 |  | 0.58 | 0.48 | 0.68 | 0.33 | 0.22 | 0.42 | 0.10 | 0.09 | 0.11 |
| 3 | 0.62 | 0.52 | 0.73 | 0.29 | 0.18 | 0.38 | 0.09 | 0.08 | 0.11 |  | 0.65 | 0.55 | 0.77 | 0.26 | 0.14 | 0.36 | 0.09 | 0.08 | 0.11 |
| 4 | 0.67 | 0.51 | 0.85 | 0.25 | 0.06 | 0.40 | 0.09 | 0.07 | 0.11 |  | 0.56 | 0.44 | 0.71 | 0.38 | 0.24 | 0.50 | 0.06 | 0.04 | 0.07 |
| 5 | 0.70 | 0.58 | 0.84 | 0.25 | 0.11 | 0.36 | 0.05 | 0.04 | 0.06 |  | 0.66 | 0.54 | 0.80 | 0.27 | 0.13 | 0.38 | 0.07 | 0.06 | 0.09 |
| 6 | 0.47 | 0.31 | 0.67 | 0.45 | 0.25 | 0.60 | 0.08 | 0.06 | 0.12 |  | 0.70 | 0.51 | 0.95 | 0.24 | 0.00 | 0.44 | 0.06 | 0.04 | 0.08 |
| 7 | 0.58 | 0.48 | 0.69 | 0.37 | 0.26 | 0.47 | 0.05 | 0.04 | 0.06 |  | 0.76 | 0.63 | 0.91 | 0.17 | 0.04 | 0.30 | 0.07 | 0.06 | 0.08 |
| 8 | 0.71 | 0.56 | 0.90 | 0.22 | 0.03 | 0.37 | 0.07 | 0.06 | 0.08 |  | 0.69 | 0.55 | 0.87 | 0.25 | 0.07 | 0.40 | 0.05 | 0.05 | 0.07 |
| 9 | 0.84 | 0.66 | 0.96 | 0.11 | 0.00 | 0.28 | 0.05 | 0.04 | 0.06 |  | 0.68 | 0.52 | 0.87 | 0.24 | 0.05 | 0.40 | 0.08 | 0.06 | 0.10 |
| 10 | 0.70 | 0.59 | 0.83 | 0.24 | 0.11 | 0.35 | 0.06 | 0.05 | 0.07 |  | 0.61 | 0.50 | 0.74 | 0.33 | 0.20 | 0.44 | 0.06 | 0.05 | 0.07 |
| 11 | 0.60 | 0.43 | 0.80 | 0.26 | 0.06 | 0.42 | 0.14 | 0.12 | 0.18 |  | 0.93 | 0.91 | 0.94 | 0.00 | 0.00 | 0.14 | 0.07 | 0.06 | 0.09 |
| 12 | 0.73 | 0.60 | 0.87 | 0.19 | 0.04 | 0.31 | 0.09 | 0.07 | 0.10 |  | 0.81 | 0.68 | 0.94 | 0.13 | 0.00 | 0.26 | 0.06 | 0.06 | 0.08 |
| 13 | 0.63 | 0.48 | 0.82 | 0.29 | 0.10 | 0.44 | 0.08 | 0.06 | 0.10 |  | 0.75 | 0.57 | 0.93 | 0.17 | 0.00 | 0.35 | 0.08 | 0.06 | 0.10 |
| 14 | 0.80 | 0.64 | 0.92 | 0.10 | 0.00 | 0.26 | 0.10 | 0.08 | 0.12 |  | 0.67 | 0.53 | 0.84 | 0.21 | 0.04 | 0.34 | 0.12 | 0.10 | 0.14 |
| 15 | 0.56 | 0.39 | 0.78 | 0.35 | 0.12 | 0.51 | 0.09 | 0.07 | 0.12 |  | 0.83 | 0.61 | 0.92 | 0.08 | 0.00 | 0.29 | 0.10 | 0.08 | 0.12 |
| 16 | 0.81 | 0.62 | 0.92 | 0.09 | 0.00 | 0.28 | 0.10 | 0.08 | 0.12 |  | 0.68 | 0.50 | 0.89 | 0.19 | 0.00 | 0.37 | 0.13 | 0.10 | 0.16 |
| 17 | 0.78 | 0.63 | 0.93 | 0.14 | 0.00 | 0.29 | 0.08 | 0.07 | 0.10 |  | 0.61 | 0.45 | 0.79 | 0.24 | 0.06 | 0.39 | 0.15 | 0.13 | 0.18 |
| 18 | 0.70 | 0.55 | 0.89 | 0.20 | 0.01 | 0.36 | 0.10 | 0.08 | 0.12 |  | 0.72 | 0.54 | 0.90 | 0.17 | 0.00 | 0.34 | 0.12 | 0.09 | 0.15 |
| 19 | 0.77 | 0.51 | 0.89 | 0.09 | 0.00 | 0.35 | 0.14 | 0.10 | 0.18 |  | 0.76 | 0.55 | 0.90 | 0.12 | 0.00 | 0.32 | 0.12 | 0.10 | 0.15 |
| 20-69 | 0.52 | 0.46 | 0.59 | 0.33 | 0.26 | 0.39 | 0.15 | 0.14 | 0.17 |  | 0.61 | 0.55 | 0.67 | 0.25 | 0.19 | 0.31 | 0.14 | 0.13 | 0.15 |

LL: lower limit of confidence interval; UL: upper limit

Supplementary table 4. Additive genetic, shared environmental and unique environmental variances of height with 95% confidence intervals by age, sex, parental education and cultural-geographic region.

|  | Additive genetic  variance | | | Shared environmental  variance | | | Unique environmental  variance | | |  | Additive genetic  variance | | | Shared environmental  variance | | | Unique environmental  variance | | |
| --- | --- | --- | --- | --- | --- | --- | --- | --- | --- | --- | --- | --- | --- | --- | --- | --- | --- | --- | --- |
| Age | a^2^ | LL | UL | c^2^ | LL | UL | e^2^ | LL | UL |  | a^2^ | LL | UL | c^2^ | LL | UL | e^2^ | LL | UL |
| European males with low parental education | | | | | | | | | |  | European females with low parental education | | | | | | | | |
| 1 | 3.60 | 3.01 | 4.24 | 3.44 | 3.26 | 4.09 | 0.95 | 0.85 | 1.07 |  | 4.16 | 3.50 | 4.89 | 2.72 | 1.97 | 3.42 | 1.06 | 0.96 | 1.18 |
| 2 | 6.77 | 5.73 | 7.95 | 3.79 | 2.58 | 4.93 | 1.28 | 1.13 | 1.45 |  | 7.12 | 6.01 | 8.38 | 3.61 | 0.77 | 4.82 | 1.22 | 1.09 | 1.38 |
| 3 | 10.00 | 8.43 | 11.81 | 3.13 | 2.02 | 4.82 | 1.52 | 1.34 | 1.74 |  | 11.31 | 9.74 | 13.13 | 2.50 | 1.02 | 4.19 | 1.22 | 1.08 | 1.38 |
| 4 | 5.76 | 2.51 | 10.11 | 9.71 | 5.14 | 13.60 | 2.80 | 2.20 | 3.64 |  | 12.22 | 8.30 | 16.82 | 3.12 | 0.00 | 7.45 | 1.95 | 1.55 | 2.51 |
| 5 | 17.60 | 14.12 | 21.95 | 7.07 | 2.61 | 11.09 | 1.74 | 1.46 | 2.10 |  | 18.28 | 14.68 | 22.82 | 8.58 | 3.82 | 12.82 | 1.77 | 1.50 | 2.12 |
| 6 | 15.23 | 7.67 | 24.32 | 5.26 | 0.00 | 13.82 | 2.83 | 1.95 | 4.31 |  | 19.98 | 12.97 | 24.62 | 0.00 | 0.00 | 7.89 | 1.60 | 1.13 | 2.37 |
| 7 | 24.00 | 19.91 | 29.03 | 4.71 | 0.00 | 9.28 | 1.36 | 1.14 | 1.64 |  | 21.32 | 17.36 | 26.17 | 7.48 | 6.99 | 11.96 | 2.25 | 1.92 | 2.67 |
| 8 | 20.31 | 15.68 | 26.34 | 11.91 | 8.85 | 17.61 | 1.89 | 1.53 | 2.36 |  | 18.79 | 14.12 | 24.99 | 10.27 | 5.23 | 15.84 | 2.11 | 1.75 | 2.58 |
| 9 | 23.63 | 15.64 | 34.97 | 14.37 | 5.40 | 23.91 | 3.95 | 3.15 | 5.04 |  | 19.80 | 14.08 | 27.83 | 20.53 | 16.57 | 28.63 | 2.16 | 1.69 | 2.83 |
| 10 | 36.09 | 29.57 | 43.90 | 6.16 | 0.00 | 13.30 | 2.34 | 1.96 | 2.81 |  | 30.56 | 24.50 | 37.99 | 8.89 | 3.21 | 15.53 | 3.84 | 3.29 | 4.52 |
| 11 | 34.39 | 26.74 | 44.27 | 9.99 | 0.00 | 18.72 | 3.43 | 2.83 | 4.21 |  | 42.39 | 32.83 | 53.00 | 6.92 | 0.00 | 17.16 | 4.41 | 3.65 | 5.40 |
| 12 | 44.79 | 36.36 | 52.25 | 3.88 | 0.00 | 12.89 | 3.46 | 2.93 | 4.11 |  | 47.69 | 39.20 | 51.92 | 0.00 | 0.00 | 8.78 | 4.73 | 4.07 | 5.55 |
| 13 | 51.62 | 37.43 | 72.28 | 22.49 | 8.39 | 40.24 | 2.55 | 1.99 | 3.34 |  | 31.41 | 23.74 | 41.64 | 14.18 | 5.06 | 23.57 | 2.58 | 2.03 | 3.34 |
| 14 | 68.63 | 55.25 | 77.66 | 2.85 | 0.00 | 16.98 | 6.09 | 5.15 | 7.28 |  | 31.64 | 24.77 | 37.62 | 3.28 | 0.00 | 10.44 | 5.02 | 4.31 | 5.89 |
| 15 | 56.93 | 38.46 | 69.39 | 2.21 | 0.00 | 21.87 | 7.34 | 5.54 | 10.01 |  | 26.53 | 18.00 | 37.22 | 7.30 | 0.00 | 17.01 | 3.59 | 2.80 | 4.71 |
| 16 | 25.80 | 17.47 | 37.88 | 19.63 | 18.25 | 30.29 | 4.55 | 3.61 | 5.86 |  | 25.36 | 17.86 | 35.30 | 8.32 | 0.00 | 16.71 | 4.04 | 3.26 | 5.09 |
| 17 | 33.97 | 24.48 | 43.58 | 5.84 | 0.00 | 16.00 | 5.61 | 4.60 | 6.93 |  | 21.48 | 15.35 | 27.28 | 10.04 | 7.92 | 16.76 | 4.94 | 4.12 | 5.98 |
| 18 | 44.10 | 32.82 | 54.37 | 4.55 | 0.00 | 17.06 | 2.35 | 1.82 | 3.11 |  | 28.82 | 20.62 | 37.46 | 5.25 | 0.00 | 14.04 | 4.72 | 3.81 | 5.95 |
| 19 | 34.01 | 20.12 | 46.76 | 4.68 | 0.00 | 20.31 | 4.74 | 3.42 | 6.85 |  | 23.80 | 14.66 | 34.78 | 6.94 | 0.00 | 17.09 | 5.01 | 3.83 | 6.71 |
| 20-69 | 29.20 | 24.05 | 35.20 | 7.11 | 3.77 | 12.61 | 4.98 | 4.33 | 5.75 |  | 27.50 | 23.48 | 32.11 | 3.71 | 0.00 | 8.09 | 3.15 | 2.80 | 3.57 |
| European males with intermediate parental education | | | | | | | | | |  | European females with intermediate parental education | | | | | | | | |
| 1 | 3.38 | 2.88 | 3.93 | 2.82 | 2.25 | 3.35 | 1.07 | 0.98 | 1.18 |  | 3.01 | 2.53 | 3.54 | 3.03 | 2.48 | 3.56 | 1.08 | 0.99 | 1.18 |
| 2 | 6.95 | 6.07 | 7.92 | 2.41 | 2.21 | 3.34 | 1.12 | 1.01 | 1.24 |  | 7.99 | 6.97 | 9.12 | 1.32 | 0.38 | 2.37 | 1.14 | 1.04 | 1.26 |
| 3 | 9.86 | 8.63 | 11.26 | 3.46 | 3.01 | 4.81 | 1.22 | 1.09 | 1.37 |  | 9.33 | 8.11 | 10.72 | 3.50 | 2.59 | 4.82 | 1.42 | 1.29 | 1.57 |
| 4 | 10.99 | 7.53 | 15.88 | 5.70 | 1.07 | 10.07 | 1.18 | 0.84 | 1.73 |  | 13.84 | 11.22 | 18.29 | 1.93 | 0.00 | 6.99 | 1.21 | 0.89 | 1.69 |
| 5 | 18.40 | 15.71 | 21.60 | 5.25 | 3.12 | 8.27 | 1.34 | 1.16 | 1.55 |  | 15.86 | 13.26 | 18.98 | 6.98 | 5.35 | 9.93 | 1.63 | 1.42 | 1.87 |
| 6 | 16.67 | 10.06 | 24.00 | 2.75 | 0.00 | 10.76 | 1.35 | 0.82 | 2.43 |  | 17.62 | 11.01 | 24.53 | 2.40 | 0.00 | 10.36 | 0.92 | 0.62 | 1.45 |
| 7 | 18.55 | 15.90 | 20.99 | 9.95 | 8.11 | 13.07 | 1.59 | 1.40 | 1.82 |  | 20.24 | 17.19 | 23.81 | 6.79 | 3.89 | 10.15 | 2.34 | 2.08 | 2.64 |
| 8 | 23.89 | 19.46 | 29.45 | 5.75 | 0.51 | 10.76 | 1.56 | 1.31 | 1.89 |  | 25.15 | 20.55 | 29.56 | 2.40 | 0.00 | 7.34 | 1.57 | 1.32 | 1.89 |
| 9 | 18.75 | 12.94 | 27.07 | 16.42 | 11.90 | 24.37 | 2.28 | 1.75 | 3.04 |  | 27.19 | 18.85 | 39.21 | 9.67 | 0.00 | 19.52 | 1.77 | 1.38 | 2.31 |
| 10 | 32.11 | 27.49 | 37.55 | 6.64 | 2.26 | 11.67 | 2.47 | 2.17 | 2.84 |  | 28.93 | 24.39 | 34.31 | 10.72 | 5.17 | 15.76 | 3.29 | 2.93 | 3.72 |
| 11 | 36.44 | 27.88 | 45.00 | 4.54 | 0.00 | 14.12 | 2.75 | 2.17 | 3.55 |  | 39.58 | 30.04 | 52.36 | 10.88 | 0.00 | 21.85 | 2.72 | 2.19 | 3.44 |
| 12 | 45.40 | 38.72 | 51.97 | 4.04 | 0.00 | 11.07 | 3.42 | 3.00 | 3.92 |  | 45.35 | 37.95 | 53.75 | 6.58 | 0.00 | 14.51 | 4.43 | 3.89 | 5.07 |
| 13 | 47.92 | 36.05 | 63.35 | 18.04 | 7.44 | 31.70 | 5.20 | 4.07 | 6.79 |  | 51.03 | 42.15 | 57.38 | 0.00 | 0.00 | 8.97 | 2.46 | 1.99 | 3.10 |
| 14 | 62.29 | 48.03 | 75.28 | 6.75 | 0.00 | 22.00 | 6.87 | 5.66 | 8.45 |  | 31.77 | 25.34 | 38.46 | 4.13 | 0.00 | 11.04 | 3.41 | 2.88 | 4.08 |
| 15 | 44.99 | 31.21 | 62.19 | 13.46 | 0.00 | 28.46 | 8.17 | 6.45 | 10.54 |  | 28.83 | 21.10 | 38.06 | 6.74 | 0.00 | 15.21 | 3.61 | 2.97 | 4.44 |
| 16 | 43.73 | 33.92 | 52.52 | 4.42 | 0.00 | 14.50 | 6.16 | 5.07 | 7.59 |  | 30.47 | 24.76 | 35.11 | 1.90 | 0.00 | 7.84 | 3.32 | 2.81 | 3.94 |
| 17 | 37.63 | 30.16 | 45.06 | 4.45 | 0.00 | 12.17 | 5.04 | 4.24 | 6.05 |  | 26.98 | 21.73 | 33.16 | 4.97 | 0.00 | 10.50 | 3.79 | 3.28 | 4.40 |
| 18 | 34.46 | 23.00 | 45.17 | 5.47 | 0.00 | 17.08 | 7.11 | 5.43 | 9.56 |  | 30.85 | 22.84 | 36.53 | 1.71 | 0.00 | 10.16 | 3.63 | 2.96 | 4.51 |
| 19 | 36.34 | 24.53 | 42.69 | 0.40 | 0.00 | 12.40 | 5.72 | 4.36 | 7.69 |  | 29.47 | 21.74 | 33.63 | 0.16 | 0.00 | 8.16 | 3.80 | 3.07 | 4.79 |
| 20-69 | 31.58 | 27.63 | 36.06 | 4.62 | 0.89 | 8.83 | 3.24 | 2.89 | 3.66 |  | 24.56 | 21.26 | 27.70 | 1.82 | 0.00 | 5.17 | 4.47 | 4.06 | 4.94 |
| European males with high parental education | | | | | | | | | |  | European females with high parental education | | | | | | | | |
| 1 | 4.06 | 3.30 | 4.90 | 2.29 | 1.44 | 3.08 | 1.11 | 0.98 | 1.26 |  | 3.99 | 3.25 | 4.82 | 2.34 | 1.52 | 3.11 | 1.18 | 1.05 | 1.32 |
| 2 | 7.28 | 6.14 | 8.59 | 2.03 | 1.26 | 3.23 | 1.17 | 1.03 | 1.34 |  | 5.87 | 4.89 | 6.92 | 3.29 | 2.66 | 4.38 | 1.03 | 0.91 | 1.18 |
| 3 | 9.32 | 7.77 | 11.12 | 2.98 | 1.84 | 4.64 | 1.40 | 1.22 | 1.62 |  | 8.36 | 6.94 | 10.01 | 3.52 | 0.08 | 5.06 | 1.33 | 1.17 | 1.53 |
| 4 | 11.00 | 7.59 | 15.56 | 3.98 | 0.00 | 7.84 | 1.73 | 1.33 | 2.31 |  | 11.37 | 8.40 | 15.09 | 2.61 | 0.00 | 5.96 | 1.15 | 0.90 | 1.50 |
| 5 | 16.48 | 13.52 | 20.13 | 4.88 | 2.36 | 8.23 | 1.30 | 1.10 | 1.57 |  | 17.23 | 13.89 | 21.33 | 5.13 | 1.72 | 8.81 | 2.00 | 1.71 | 2.35 |
| 6 | 18.63 | 11.89 | 27.90 | 5.38 | 0.00 | 13.53 | 1.72 | 1.11 | 2.89 |  | 16.26 | 9.65 | 24.97 | 5.06 | 0.00 | 13.32 | 1.88 | 1.33 | 2.79 |
| 7 | 18.87 | 15.58 | 22.90 | 8.20 | 6.15 | 12.10 | 1.51 | 1.27 | 1.81 |  | 22.76 | 18.61 | 27.88 | 4.58 | 0.00 | 9.11 | 1.86 | 1.60 | 2.17 |
| 8 | 22.93 | 17.28 | 30.07 | 5.76 | 0.00 | 12.02 | 2.74 | 2.20 | 3.46 |  | 25.03 | 19.40 | 30.34 | 2.78 | 0.00 | 8.88 | 1.67 | 1.37 | 2.06 |
| 9 | 29.48 | 21.17 | 36.13 | 2.36 | 0.00 | 11.64 | 1.66 | 1.29 | 2.19 |  | 28.60 | 19.27 | 33.05 | 0.00 | 0.00 | 10.15 | 2.95 | 2.31 | 3.83 |
| 10 | 30.03 | 25.00 | 36.16 | 10.03 | 8.08 | 15.79 | 2.25 | 1.91 | 2.69 |  | 24.92 | 20.06 | 30.97 | 12.67 | 9.58 | 18.33 | 2.45 | 2.10 | 2.88 |
| 11 | 28.02 | 20.44 | 37.87 | 9.83 | 0.08 | 18.31 | 4.13 | 3.25 | 5.34 |  | 44.83 | 34.62 | 50.43 | 0.00 | 0.00 | 10.65 | 3.64 | 2.93 | 4.61 |
| 12 | 37.11 | 30.53 | 45.07 | 8.03 | 0.28 | 15.17 | 3.66 | 3.10 | 4.36 |  | 42.41 | 34.72 | 50.22 | 4.86 | 0.00 | 13.05 | 3.61 | 3.10 | 4.24 |
| 13 | 42.87 | 30.21 | 60.35 | 17.58 | 0.00 | 32.51 | 5.09 | 3.88 | 6.86 |  | 24.87 | 16.80 | 36.17 | 14.20 | 5.22 | 23.78 | 3.63 | 2.84 | 4.74 |
| 14 | 52.50 | 40.67 | 64.32 | 7.24 | 0.00 | 19.75 | 7.55 | 6.31 | 9.14 |  | 26.21 | 20.02 | 33.72 | 6.60 | 0.00 | 13.10 | 5.18 | 4.39 | 6.16 |
| 15 | 32.73 | 19.18 | 51.99 | 26.13 | 8.90 | 42.38 | 7.86 | 5.96 | 10.67 |  | 33.59 | 23.44 | 40.24 | 1.47 | 0.00 | 12.54 | 3.55 | 2.77 | 4.65 |
| 16 | 29.29 | 19.07 | 43.00 | 12.62 | 0.00 | 23.92 | 5.70 | 4.35 | 7.66 |  | 20.15 | 13.67 | 28.87 | 7.44 | 0.00 | 14.75 | 3.56 | 2.79 | 4.63 |
| 17 | 41.99 | 32.63 | 48.30 | 1.54 | 0.00 | 11.45 | 3.66 | 3.00 | 4.52 |  | 23.04 | 15.77 | 31.33 | 6.08 | 0.00 | 13.58 | 6.57 | 5.50 | 7.93 |
| 18 | 28.53 | 19.44 | 38.40 | 5.78 | 0.00 | 15.77 | 4.13 | 3.17 | 5.53 |  | 26.37 | 17.71 | 34.75 | 4.32 | 0.00 | 13.43 | 5.63 | 4.43 | 7.29 |
| 19 | 43.02 | 26.72 | 53.05 | 0.00 | 0.00 | 16.71 | 5.91 | 4.03 | 9.17 |  | 25.71 | 17.01 | 32.26 | 1.54 | 0.00 | 10.82 | 3.52 | 2.69 | 4.74 |
| 20-69 | 29.98 | 24.08 | 37.15 | 7.09 | 0.00 | 13.59 | 3.89 | 3.29 | 4.64 |  | 27.37 | 22.44 | 33.19 | 6.29 | 2.82 | 11.59 | 4.06 | 3.51 | 4.74 |
|  |  |  |  |  |  |  |  |  |  |  |  |  |  |  |  |  |  |  |  |
| North-American and Australian males with low parental education | | | | | | | | | |  | North-American and Australian females with low parental education | | | | | | | | |
| 1 |  |  |  |  |  |  |  |  |  |  |  |  |  |  |  |  |  |  |  |
| 2 | 16.44 | 10.47 | 26.49 | 0.00 | 0.00 | 4.32 | 0.95 | 0.48 | 2.39 |  | 4.76 | 0.00 | 14.91 | 5.65 | 0.00 | 14.89 | 1.93 | 1.03 | 4.29 |
| 3 | 14.30 | 7.84 | 24.46 | 16.28 | 5.38 | 25.87 | 2.37 | 1.61 | 3.71 |  | 8.85 | 1.25 | 21.68 | 16.53 | 4.53 | 26.45 | 8.59 | 5.95 | 12.24 |
| 4 | 15.96 | 11.18 | 22.49 | 26.78 | 18.76 | 34.79 | 2.15 | 1.61 | 2.97 |  | 12.47 | 5.41 | 21.50 | 22.06 | 12.53 | 30.54 | 5.37 | 4.00 | 7.45 |
| 5 | 48.59 | 33.50 | 60.50 | 6.77 | 0.00 | 24.30 | 1.80 | 1.20 | 2.89 |  | 28.46 | 16.12 | 50.81 | 34.21 | 12.21 | 51.92 | 5.77 | 3.86 | 9.01 |
| 6 | 20.78 | 9.24 | 40.17 | 64.48 | 40.52 | 89.51 | 5.50 | 3.70 | 8.62 |  | 51.48 | 32.20 | 67.77 | 8.39 | 0.33 | 29.89 | 4.40 | 2.68 | 7.97 |
| 7 | 24.11 | 12.08 | 44.90 | 54.75 | 31.07 | 78.49 | 3.67 | 2.39 | 6.08 |  | 27.73 | 13.73 | 50.39 | 41.07 | 17.08 | 63.50 | 4.95 | 3.23 | 8.18 |
| 8 | 16.08 | 4.97 | 33.80 | 45.62 | 25.61 | 66.30 | 4.19 | 2.55 | 7.61 |  | 53.49 | 29.87 | 74.75 | 7.70 | 0.00 | 35.85 | 3.98 | 2.37 | 7.44 |
| 9 | 25.87 | 15.23 | 42.09 | 32.01 | 14.39 | 47.29 | 4.47 | 3.20 | 6.51 |  | 30.53 | 15.96 | 56.65 | 26.42 | 0.00 | 45.36 | 4.92 | 3.44 | 7.38 |
| 10 | 52.50 | 32.76 | 65.78 | 0.81 | 0.00 | 23.38 | 3.96 | 2.60 | 6.49 |  | 45.24 | 27.72 | 71.47 | 18.00 | 0.00 | 40.39 | 3.47 | 2.37 | 5.35 |
| 11 | 32.10 | 0.97 | 74.98 | 37.29 | 0.00 | 69.55 | 16.73 | 10.93 | 27.37 |  | 60.85 | 36.99 | 90.73 | 14.51 | 0.00 | 43.61 | 4.54 | 2.96 | 7.51 |
| 12 | 45.79 | 23.26 | 79.48 | 27.21 | 0.00 | 54.62 | 10.17 | 7.19 | 15.05 |  | 28.95 | 9.32 | 57.44 | 31.52 | 10.94 | 55.20 | 10.50 | 7.12 | 16.40 |
| 13 | 103.81 | 54.74 | 135.55 | 0.00 | 0.00 | 49.11 | 22.12 | 15.07 | 34.08 |  | 55.96 | 27.19 | 72.91 | 0.00 | 0.00 | 29.87 | 11.64 | 7.94 | 17.94 |
| 14 | 75.85 | 53.23 | 94.92 | 0.00 | 0.00 | 23.22 | 7.29 | 5.01 | 11.15 |  | 27.78 | 12.84 | 45.25 | 9.71 | 0.00 | 25.93 | 7.12 | 4.91 | 10.88 |
| 15 | 66.81 | 43.05 | 95.17 | 12.27 | 0.00 | 41.25 | 4.22 | 2.85 | 6.63 |  | 52.17 | 35.90 | 66.29 | 0.00 | 0.00 | 17.22 | 3.16 | 2.05 | 5.24 |
| 16 | 32.31 | 11.70 | 62.15 | 24.61 | 0.00 | 48.32 | 11.46 | 8.03 | 17.10 |  | 27.87 | 16.34 | 44.88 | 16.31 | 0.31 | 30.77 | 4.80 | 3.43 | 7.02 |
| 17 | 47.50 | 31.68 | 62.03 | 5.35 | 0.00 | 23.41 | 5.58 | 4.14 | 7.76 |  | 32.26 | 20.54 | 48.38 | 10.55 | 0.00 | 25.17 | 3.22 | 2.39 | 4.50 |
| 18 | 33.54 | 24.23 | 46.21 | 10.46 | 0.00 | 21.77 | 3.86 | 3.02 | 5.04 |  | 25.13 | 12.57 | 48.59 | 22.02 | 0.00 | 40.55 | 3.56 | 2.42 | 5.52 |
| 19 | 35.32 | 20.54 | 57.23 | 14.35 | 0.00 | 33.16 | 5.05 | 3.45 | 7.81 |  | 37.83 | 25.65 | 48.05 | 0.00 | 0.00 | 13.22 | 3.51 | 2.53 | 5.07 |
| 20-69 | 35.50 | 31.68 | 39.68 | 8.19 | 6.89 | 12.14 | 6.43 | 5.96 | 6.96 |  | 30.57 | 27.39 | 34.01 | 9.96 | 6.65 | 13.26 | 6.68 | 6.26 | 7.13 |
| North-American and Australian males with intermediate parental education | | | | | | | | | |  | North-American and Australian females with intermediate parental education | | | | | | | | |
| 1 |  |  |  |  |  |  |  |  |  |  |  |  |  |  |  |  |  |  |  |
| 2 | 2.60 | 0.00 | 6.72 | 2.96 | 0.00 | 6.34 | 2.74 | 1.83 | 4.34 |  | 8.10 | 4.69 | 13.77 | 4.61 | 0.00 | 10.13 | 0.89 | 0.58 | 1.49 |
| 3 | 13.94 | 11.43 | 20.21 | 7.65 | 0.20 | 13.41 | 1.84 | 1.33 | 2.65 |  | 12.34 | 8.45 | 18.12 | 12.99 | 6.60 | 19.07 | 1.16 | 0.86 | 1.62 |
| 4 | 16.96 | 11.69 | 24.69 | 19.55 | 10.93 | 27.73 | 1.41 | 0.97 | 2.15 |  | 19.44 | 12.40 | 29.85 | 13.36 | 11.01 | 22.21 | 2.54 | 1.81 | 3.74 |
| 5 | 20.67 | 13.45 | 33.02 | 27.30 | 13.36 | 41.30 | 0.67 | 0.39 | 1.29 |  | 21.59 | 6.41 | 45.37 | 26.90 | 2.83 | 45.92 | 5.63 | 3.37 | 10.41 |
| 6 | 22.41 | 9.16 | 47.71 | 50.73 | 34.89 | 80.06 | 3.37 | 1.89 | 6.85 |  | 30.60 | 16.24 | 54.12 | 36.67 | 12.27 | 59.82 | 4.14 | 2.56 | 7.29 |
| 7 | 50.70 | 30.19 | 73.35 | 7.27 | 0.00 | 32.00 | 3.73 | 2.11 | 7.54 |  | 24.61 | 15.50 | 40.53 | 17.57 | 2.97 | 32.10 | 1.27 | 0.75 | 2.38 |
| 8 | 37.96 | 22.00 | 51.62 | 3.58 | 0.00 | 22.24 | 2.59 | 1.43 | 5.47 |  | 14.60 | 0.41 | 52.21 | 38.40 | 3.70 | 62.67 | 18.27 | 10.45 | 30.46 |
| 9 | 36.03 | 21.35 | 48.44 | 4.41 | 0.00 | 18.80 | 7.71 | 5.50 | 11.26 |  | 49.02 | 32.65 | 63.55 | 5.40 | 0.00 | 24.38 | 3.34 | 2.41 | 4.82 |
| 10 | 45.03 | 31.14 | 56.10 | 0.00 | 0.00 | 15.12 | 2.70 | 1.72 | 4.57 |  | 27.55 | 12.48 | 52.14 | 29.67 | 8.02 | 49.96 | 5.23 | 3.28 | 9.08 |
| 11 | 33.17 | 16.91 | 58.20 | 16.33 | 0.00 | 37.66 | 4.03 | 2.47 | 7.23 |  | 57.01 | 30.26 | 86.20 | 10.21 | 0.00 | 43.33 | 3.14 | 1.93 | 5.60 |
| 12 | 68.02 | 37.35 | 103.82 | 15.09 | 0.00 | 51.37 | 8.73 | 5.90 | 13.69 |  | 40.74 | 7.22 | 66.35 | 8.76 | 0.00 | 40.56 | 21.01 | 14.59 | 31.64 |
| 13 | 28.52 | 6.56 | 60.79 | 55.46 | 21.86 | 87.56 | 10.22 | 6.52 | 17.31 |  | 30.38 | 12.77 | 48.00 | 7.15 | 0.00 | 26.27 | 6.93 | 4.66 | 10.97 |
| 14 | 83.05 | 38.88 | 105.57 | 0.00 | 0.00 | 48.30 | 11.21 | 7.38 | 18.24 |  | 11.06 | 0.00 | 31.78 | 17.52 | 0.00 | 32.29 | 10.94 | 7.36 | 17.05 |
| 15 | 75.05 | 52.37 | 94.56 | 0.00 | 0.00 | 24.00 | 6.07 | 3.92 | 10.11 |  | 35.62 | 18.64 | 46.26 | 0.62 | 0.00 | 19.65 | 4.94 | 3.27 | 7.91 |
| 16 | 40.90 | 26.45 | 64.47 | 22.82 | 0.00 | 43.30 | 2.97 | 2.08 | 4.45 |  | 44.50 | 35.05 | 54.48 | 0.00 | 0.00 | 8.62 | 4.43 | 3.25 | 6.27 |
| 17 | 48.15 | 30.87 | 63.14 | 4.08 | 0.00 | 24.78 | 4.38 | 3.22 | 6.18 |  | 31.12 | 19.46 | 47.02 | 11.06 | 0.00 | 25.17 | 4.67 | 3.58 | 6.25 |
| 18 | 49.42 | 40.01 | 60.21 | 0.00 | 0.00 | 7.33 | 6.45 | 4.81 | 8.93 |  | 24.38 | 4.66 | 42.96 | 8.49 | 0.00 | 26.80 | 12.29 | 8.46 | 18.77 |
| 19 | 30.48 | 17.11 | 55.74 | 21.42 | 0.00 | 42.28 | 2.57 | 1.82 | 3.79 |  | 11.99 | 0.00 | 40.48 | 31.98 | 4.07 | 55.56 | 12.88 | 8.55 | 20.39 |
| 20-69 | 30.64 | 27.29 | 34.32 | 11.83 | 10.59 | 15.42 | 5.90 | 5.48 | 6.36 |  | 30.58 | 27.57 | 33.85 | 8.60 | 6.38 | 11.75 | 5.44 | 5.09 | 5.82 |
| North-American and Australian males with high parental education | | | | | | | | | |  | North-American and Australian females with high parental education | | | | | | | | |
| 1 |  |  |  |  |  |  |  |  |  |  |  |  |  |  |  |  |  |  |  |
| 2 | 2.64 | 0.00 | 8.63 | 6.71 | 0.00 | 14.05 | 1.24 | 0.65 | 2.78 |  | 7.07 | 4.69 | 11.07 | 0.00 | 0.00 | 5.04 | 0.46 | 0.24 | 1.03 |
| 3 | 10.86 | 6.71 | 17.38 | 14.24 | 7.31 | 21.54 | 1.33 | 0.85 | 2.28 |  | 17.41 | 14.16 | 25.72 | 5.22 | 0.00 | 12.95 | 1.25 | 0.87 | 1.91 |
| 4 | 24.42 | 16.16 | 34.23 | 6.29 | 0.00 | 15.44 | 3.05 | 2.12 | 4.61 |  | 16.00 | 10.42 | 24.21 | 18.59 | 11.56 | 26.84 | 1.88 | 1.31 | 2.82 |
| 5 | 30.17 | 28.84 | 47.36 | 13.18 | 0.00 | 28.26 | 1.70 | 1.08 | 2.92 |  | 19.40 | 10.86 | 30.99 | 22.18 | 12.76 | 35.72 | 1.89 | 1.06 | 3.86 |
| 6 | 22.29 | 7.64 | 46.73 | 38.70 | 14.05 | 60.01 | 7.58 | 4.78 | 12.79 |  | 51.25 | 31.85 | 66.58 | 4.96 | 0.00 | 28.83 | 2.25 | 1.27 | 4.57 |
| 7 | 34.15 | 19.53 | 60.09 | 44.40 | 35.96 | 70.20 | 2.79 | 1.69 | 5.06 |  | 34.35 | 19.94 | 52.74 | 11.42 | 0.00 | 29.91 | 3.18 | 1.85 | 6.18 |
| 8 | 36.21 | 19.13 | 67.58 | 23.15 | 0.00 | 48.26 | 2.49 | 1.52 | 4.47 |  | 33.13 | 14.74 | 64.27 | 30.41 | 0.00 | 55.97 | 5.06 | 2.86 | 10.22 |
| 9 | 40.71 | 28.03 | 55.71 | 8.40 | 0.00 | 23.26 | 2.83 | 1.99 | 4.23 |  | 25.87 | 16.98 | 38.45 | 22.18 | 11.60 | 34.03 | 3.44 | 2.46 | 5.04 |
| 10 | 41.54 | 40.41 | 56.99 | 5.49 | 0.00 | 23.02 | 3.46 | 2.26 | 5.66 |  | 31.78 | 17.44 | 59.72 | 34.14 | 5.93 | 57.35 | 2.57 | 1.63 | 4.40 |
| 11 | 20.45 | 0.00 | 53.97 | 19.79 | 0.00 | 47.66 | 19.94 | 12.26 | 34.41 |  | 48.27 | 28.34 | 66.89 | 5.74 | 0.00 | 28.85 | 3.87 | 2.36 | 6.97 |
| 12 | 54.64 | 25.95 | 71.58 | 0.00 | 0.00 | 29.00 | 13.01 | 8.78 | 20.23 |  | 65.31 | 43.58 | 89.86 | 9.38 | 0.00 | 35.49 | 4.24 | 2.75 | 7.01 |
| 13 | 63.14 | 37.19 | 96.15 | 16.65 | 0.00 | 48.29 | 6.71 | 4.14 | 11.92 |  | 59.62 | 35.53 | 77.10 | 0.00 | 0.00 | 27.25 | 4.28 | 2.72 | 7.32 |
| 14 | 63.94 | 38.01 | 81.73 | 2.59 | 0.00 | 32.25 | 4.95 | 3.31 | 7.86 |  | 32.93 | 18.20 | 55.99 | 16.54 | 0.00 | 35.55 | 3.57 | 2.37 | 5.78 |
| 15 | 50.49 | 29.77 | 78.16 | 15.48 | 0.00 | 41.24 | 3.93 | 2.65 | 6.17 |  | 27.68 | 7.69 | 52.23 | 15.85 | 0.00 | 36.69 | 8.24 | 5.05 | 14.74 |
| 16 | 57.92 | 45.04 | 71.47 | 0.00 | 0.00 | 12.25 | 4.55 | 3.14 | 6.96 |  | 29.03 | 13.83 | 45.94 | 8.96 | 0.00 | 25.15 | 7.45 | 5.08 | 11.59 |
| 17 | 35.06 | 23.52 | 51.79 | 19.18 | 4.65 | 35.08 | 4.29 | 3.08 | 6.23 |  | 25.18 | 15.07 | 40.57 | 16.70 | 3.09 | 30.31 | 3.84 | 2.70 | 5.71 |
| 18 | 32.70 | 23.36 | 45.48 | 10.00 | 0.00 | 21.13 | 3.70 | 2.90 | 4.83 |  | 44.98 | 29.05 | 67.16 | 12.77 | 0.00 | 33.91 | 2.39 | 1.55 | 3.96 |
| 19 | 20.89 | 11.25 | 40.66 | 12.61 | 0.00 | 30.17 | 6.87 | 4.83 | 10.23 |  | 24.76 | 11.23 | 40.97 | 8.27 | 0.00 | 23.58 | 5.88 | 4.05 | 8.99 |
| 20-69 | 23.08 | 19.66 | 26.80 | 17.85 | 14.05 | 21.47 | 8.48 | 7.86 | 9.15 |  | 26.16 | 23.00 | 29.61 | 13.29 | 6.98 | 16.64 | 7.00 | 6.54 | 7.51 |
|  |  |  |  |  |  |  |  |  |  |  |  |  |  |  |  |  |  |  |  |
| East-Asian males with low parental education | | | | | | | | | |  | East-Asian females with low parental education | | | | | | | | |
| 1 | 3.24 | 0.16 | 8.63 | 6.70 | 1.31 | 11.95 | 1.12 | 0.69 | 2.01 |  | 1.78 | 0.00 | 7.04 | 9.22 | 3.34 | 14.43 | 0.98 | 0.62 | 1.67 |
| 2 | 3.50 | 0.65 | 6.41 | 0.65 | 0.00 | 3.64 | 1.05 | 0.60 | 2.01 |  | 3.02 | 0.55 | 8.19 | 3.70 | 0.00 | 7.82 | 0.76 | 0.42 | 1.54 |
| 3 | 12.34 | 4.89 | 18.09 | 1.22 | 0.00 | 10.87 | 0.93 | 0.60 | 1.55 |  | 8.35 | 2.07 | 15.83 | 3.74 | 0.00 | 10.73 | 3.12 | 1.98 | 5.24 |
| 4 | 1.06 | 0.00 | 9.10 | 13.13 | 4.85 | 23.91 | 3.89 | 2.14 | 7.17 |  | 1.05 | 0.00 | 8.22 | 14.32 | 5.82 | 24.90 | 0.87 | 0.47 | 1.80 |
| 5 | 14.34 | 4.47 | 29.94 | 9.58 | 0.00 | 22.85 | 3.06 | 1.88 | 5.22 |  | 12.67 | 5.17 | 23.02 | 5.05 | 0.00 | 14.96 | 1.83 | 1.12 | 3.29 |
| 6 | 5.49 | 0.00 | 19.24 | 17.16 | 3.91 | 29.40 | 3.53 | 2.04 | 6.68 |  | 3.93 | 0.34 | 19.14 | 13.91 | 0.00 | 24.06 | 1.01 | 0.61 | 1.86 |
| 7 | 21.23 | 8.06 | 29.91 | 0.32 | 0.00 | 14.44 | 4.59 | 3.05 | 7.33 |  | 18.14 | 8.87 | 33.05 | 6.95 | 0.00 | 20.42 | 1.41 | 0.87 | 2.53 |
| 8 | 16.11 | 7.78 | 31.55 | 26.53 | 7.63 | 46.92 | 1.35 | 0.74 | 2.91 |  | 20.62 | 8.17 | 45.60 | 14.30 | 0.00 | 34.84 | 1.38 | 0.80 | 2.65 |
| 9 | 13.01 | 3.47 | 29.00 | 18.37 | 2.59 | 33.12 | 3.69 | 2.32 | 6.44 |  | 12.93 | 5.53 | 26.69 | 20.66 | 6.48 | 35.31 | 2.11 | 1.32 | 3.71 |
| 10 | 47.84 | 29.10 | 64.42 | 1.27 | 0.00 | 24.83 | 0.84 | 0.54 | 1.41 |  | 46.03 | 26.04 | 64.17 | 0.00 | 0.00 | 23.89 | 2.03 | 1.25 | 3.61 |
| 11 | 13.19 | 5.60 | 26.21 | 25.74 | 11.61 | 40.15 | 2.98 | 2.03 | 4.67 |  | 47.16 | 35.24 | 61.10 | 0.00 | 0.00 | 17.14 | 2.84 | 1.98 | 4.26 |
| 12 | 6.40 | 0.00 | 25.00 | 41.95 | 20.79 | 64.06 | 4.81 | 3.23 | 7.50 |  | 22.16 | 7.38 | 52.95 | 25.98 | 0.00 | 52.05 | 5.29 | 3.39 | 8.90 |
| 13 | 46.42 | 20.39 | 96.27 | 31.52 | 0.00 | 75.46 | 4.41 | 2.69 | 8.02 |  | 19.92 | 7.08 | 39.39 | 10.77 | 0.00 | 27.05 | 4.56 | 2.97 | 7.51 |
| 14 | 0.00 | 0.00 | 10.29 | 54.30 | 36.17 | 83.46 | 7.80 | 5.26 | 12.26 |  | 4.25 | 0.00 | 20.18 | 18.05 | 4.34 | 34.89 | 6.59 | 3.58 | 13.03 |
| 15 | 9.51 | 0.00 | 32.78 | 35.74 | 10.79 | 62.14 | 3.02 | 1.76 | 5.82 |  | 19.22 | 1.95 | 33.06 | 3.26 | 0.00 | 24.51 | 5.73 | 3.75 | 9.35 |
| 16 | 3.28 | 0.00 | 22.94 | 35.15 | 24.21 | 61.31 | 3.08 | 1.81 | 5.72 |  | 26.50 | 9.58 | 51.96 | 10.71 | 0.00 | 39.21 | 1.02 | 0.55 | 2.19 |
| 17 | 6.43 | 0.00 | 29.33 | 23.30 | 0.00 | 42.27 | 4.76 | 3.00 | 8.01 |  | 6.51 | 0.00 | 25.16 | 10.96 | 0.00 | 25.67 | 9.81 | 6.49 | 14.42 |
| 18 | 18.71 | 7.16 | 48.53 | 24.63 | 0.00 | 49.62 | 1.98 | 1.20 | 3.61 |  | 9.55 | 2.12 | 26.25 | 12.78 | 0.00 | 25.45 | 2.05 | 1.27 | 3.63 |
| 19 | 0.33 | 0.00 | 8.49 | 31.07 | 19.97 | 45.62 | 2.50 | 1.63 | 3.92 |  | 21.97 | 9.95 | 34.53 | 4.49 | 0.00 | 20.71 | 2.27 | 1.42 | 3.94 |
| 20-69 | 28.64 | 15.98 | 40.81 | 4.27 | 0.00 | 19.32 | 2.69 | 2.05 | 3.63 |  | 15.12 | 8.82 | 25.68 | 12.07 | 1.54 | 20.26 | 2.19 | 1.81 | 2.69 |
| East-Asian males with intermediate parental education | | | | | | | | | |  | East-Asian females with intermediate parental education | | | | | | | | |
| 1 | 4.32 | 1.83 | 8.58 | 6.05 | 1.52 | 9.50 | 1.15 | 0.81 | 1.69 |  | 3.82 | 0.19 | 8.63 | 5.25 | 0.76 | 8.92 | 3.10 | 2.32 | 4.25 |
| 2 | 7.07 | 3.92 | 11.28 | 2.91 | 0.00 | 7.02 | 0.60 | 0.41 | 0.91 |  | 4.13 | 1.73 | 8.85 | 9.61 | 4.58 | 14.00 | 0.79 | 0.56 | 1.16 |
| 3 | 13.52 | 8.77 | 16.74 | 0.37 | 0.00 | 5.67 | 0.93 | 0.63 | 1.45 |  | 6.37 | 3.12 | 11.40 | 8.00 | 2.84 | 12.41 | 1.65 | 1.20 | 2.36 |
| 4 | 7.88 | 3.58 | 15.75 | 15.44 | 6.64 | 23.89 | 1.31 | 0.83 | 2.23 |  | 6.82 | 3.65 | 12.44 | 10.82 | 4.60 | 16.81 | 0.86 | 0.56 | 1.41 |
| 5 | 2.75 | 0.00 | 8.72 | 19.86 | 12.66 | 28.41 | 2.59 | 1.59 | 4.41 |  | 18.82 | 10.93 | 25.89 | 2.66 | 0.00 | 12.81 | 0.89 | 0.59 | 1.45 |
| 6 | 15.42 | 9.39 | 25.40 | 12.94 | 2.66 | 22.22 | 1.47 | 1.00 | 2.28 |  | 9.14 | 5.47 | 15.44 | 13.69 | 6.56 | 20.53 | 0.94 | 0.67 | 1.38 |
| 7 | 16.75 | 8.34 | 30.02 | 9.01 | 0.00 | 20.40 | 2.47 | 1.60 | 4.08 |  | 19.17 | 12.39 | 34.87 | 13.36 | 0.00 | 26.27 | 1.68 | 1.15 | 2.61 |
| 8 | 4.58 | 0.00 | 12.20 | 19.72 | 10.84 | 29.15 | 3.13 | 2.03 | 5.18 |  | 17.21 | 10.22 | 27.90 | 9.16 | 0.07 | 18.65 | 1.75 | 1.25 | 2.55 |
| 9 | 18.45 | 9.96 | 32.02 | 10.84 | 0.00 | 22.41 | 2.40 | 1.47 | 4.33 |  | 36.80 | 17.77 | 47.71 | 0.00 | 0.00 | 21.36 | 3.95 | 2.78 | 5.87 |
| 10 | 16.68 | 8.31 | 31.70 | 17.44 | 9.56 | 30.57 | 2.60 | 1.73 | 4.15 |  | 27.00 | 16.26 | 43.39 | 12.41 | 0.00 | 26.03 | 3.54 | 2.55 | 5.11 |
| 11 | 23.94 | 8.83 | 46.40 | 23.79 | 1.97 | 43.62 | 6.47 | 4.15 | 9.77 |  | 44.87 | 26.60 | 74.10 | 19.09 | 0.00 | 44.74 | 1.88 | 1.35 | 2.73 |
| 12 | 46.97 | 28.03 | 60.07 | 0.19 | 0.00 | 22.80 | 2.42 | 1.63 | 3.82 |  | 35.85 | 20.95 | 53.24 | 8.19 | 0.00 | 26.53 | 3.56 | 2.50 | 5.30 |
| 13 | 10.64 | 0.00 | 39.41 | 47.38 | 3.76 | 77.18 | 17.36 | 10.87 | 28.77 |  | 12.98 | 3.99 | 29.09 | 25.86 | 9.28 | 41.43 | 4.02 | 2.79 | 6.07 |
| 14 | 34.81 | 16.10 | 62.25 | 13.79 | 0.00 | 40.95 | 3.80 | 2.36 | 6.68 |  | 32.13 | 18.34 | 42.16 | 0.00 | 0.00 | 15.96 | 3.27 | 2.22 | 5.07 |
| 15 | 14.87 | 0.00 | 37.62 | 10.41 | 0.00 | 31.80 | 10.34 | 6.18 | 19.11 |  | 16.58 | 4.83 | 33.76 | 9.88 | 0.00 | 25.01 | 4.40 | 2.92 | 6.21 |
| 16 | 34.35 | 18.67 | 49.27 | 0.00 | 0.00 | 20.41 | 2.20 | 1.30 | 4.14 |  | 19.77 | 10.37 | 29.40 | 2.19 | 0.00 | 13.28 | 2.48 | 1.59 | 4.16 |
| 17 | 10.41 | 0.00 | 28.48 | 8.99 | 0.00 | 24.11 | 9.95 | 5.75 | 17.26 |  | 14.32 | 3.82 | 29.90 | 9.94 | 0.00 | 23.93 | 3.99 | 2.63 | 6.38 |
| 18 | 27.21 | 17.48 | 39.15 | 0.00 | 0.00 | 10.27 | 2.39 | 1.42 | 4.49 |  | 9.37 | 0.33 | 21.32 | 6.57 | 0.00 | 17.36 | 3.40 | 2.05 | 6.22 |
| 19 | 19.13 | 2.69 | 35.29 | 3.66 | 0.00 | 21.37 | 4.84 | 2.72 | 9.97 |  | 16.16 | 0.69 | 25.09 | 0.00 | 0.00 | 16.70 | 5.11 | 3.15 | 9.00 |
| 20-69 | 30.48 | 19.97 | 39.56 | 0.00 | 0.00 | 11.16 | 2.81 | 2.01 | 4.12 |  | 25.41 | 16.50 | 30.68 | 0.00 | 0.00 | 9.61 | 2.41 | 1.91 | 3.09 |
| East-Asian males with high parental education | | | | | | | | | |  | East-Asian females with high parental education | | | | | | | | |
| 1 | 2.76 | 0.34 | 7.31 | 4.59 | 0.40 | 8.89 | 0.88 | 0.50 | 1.74 |  | 0.25 | 0.00 | 4.02 | 7.49 | 3.55 | 12.01 | 1.51 | 0.84 | 2.93 |
| 2 | 4.77 | 0.61 | 10.49 | 2.77 | 0.00 | 8.60 | 0.82 | 0.45 | 1.77 |  | 4.27 | 0.58 | 14.12 | 8.23 | 0.00 | 15.38 | 0.75 | 0.39 | 1.71 |
| 3 | 5.94 | 2.36 | 12.98 | 9.98 | 2.89 | 16.52 | 0.99 | 0.62 | 1.71 |  | 11.70 | 3.34 | 23.61 | 6.99 | 0.00 | 18.38 | 2.03 | 1.18 | 3.99 |
| 4 | 4.60 | 0.00 | 15.87 | 19.55 | 7.35 | 32.29 | 1.60 | 0.89 | 3.32 |  | 9.55 | 4.67 | 20.60 | 12.54 | 1.72 | 24.38 | 0.39 | 0.20 | 0.94 |
| 5 | 12.19 | 6.11 | 25.20 | 14.21 | 1.04 | 26.12 | 0.91 | 0.52 | 1.77 |  | 21.99 | 10.70 | 30.69 | 0.00 | 0.00 | 13.59 | 1.76 | 1.04 | 3.38 |
| 6 | 21.65 | 10.97 | 40.77 | 11.91 | 0.00 | 29.86 | 1.36 | 0.82 | 2.51 |  | 6.29 | 0.96 | 14.96 | 23.31 | 11.79 | 38.26 | 1.63 | 0.88 | 3.51 |
| 7 | 16.03 | 7.42 | 28.89 | 7.80 | 0.00 | 19.19 | 2.41 | 1.48 | 4.32 |  | 19.73 | 8.82 | 34.60 | 7.60 | 0.00 | 21.93 | 2.40 | 1.52 | 4.10 |
| 8 | 23.46 | 14.23 | 36.02 | 6.39 | 0.00 | 19.48 | 0.71 | 0.45 | 1.19 |  | 10.28 | 4.03 | 21.86 | 29.40 | 14.74 | 46.61 | 2.05 | 1.29 | 3.55 |
| 9 | 27.30 | 15.47 | 37.59 | 0.92 | 0.00 | 14.24 | 2.25 | 1.47 | 3.67 |  | 29.11 | 17.69 | 39.86 | 0.00 | 0.00 | 12.44 | 2.85 | 1.83 | 4.79 |
| 10 | 13.21 | 6.52 | 25.52 | 19.90 | 6.93 | 32.81 | 1.77 | 1.14 | 2.96 |  | 36.07 | 19.72 | 59.34 | 12.29 | 0.00 | 34.44 | 2.87 | 1.95 | 4.46 |
| 11 | 30.09 | 12.81 | 47.51 | 6.73 | 0.00 | 27.85 | 4.72 | 3.16 | 7.28 |  | 46.65 | 35.48 | 59.98 | 0.00 | 0.00 | 10.17 | 3.14 | 2.16 | 4.84 |
| 12 | 21.88 | 10.64 | 44.95 | 42.41 | 17.65 | 65.53 | 2.49 | 1.72 | 3.80 |  | 26.95 | 13.36 | 54.91 | 30.24 | 1.68 | 53.23 | 2.87 | 1.93 | 4.56 |
| 13 | 9.88 | 0.28 | 31.26 | 47.07 | 24.25 | 70.39 | 5.63 | 3.75 | 8.90 |  | 35.15 | 20.63 | 48.82 | 4.21 | 0.00 | 21.12 | 3.44 | 2.33 | 5.36 |
| 14 | 30.94 | 16.23 | 52.46 | 9.81 | 0.00 | 31.02 | 1.69 | 1.13 | 2.71 |  | 18.06 | 7.89 | 32.51 | 7.37 | 0.00 | 20.75 | 3.01 | 1.95 | 5.00 |
| 15 | 16.17 | 3.05 | 55.25 | 30.19 | 0.00 | 55.56 | 3.40 | 2.09 | 6.06 |  | 28.94 | 19.99 | 40.06 | 0.00 | 0.00 | 8.33 | 2.06 | 1.33 | 3.44 |
| 16 | 8.92 | 0.95 | 23.76 | 17.10 | 3.43 | 30.95 | 3.46 | 2.21 | 5.81 |  | 19.81 | 8.90 | 31.94 | 2.50 | 0.00 | 15.93 | 2.77 | 1.76 | 4.73 |
| 17 | 17.13 | 0.00 | 48.91 | 15.34 | 0.00 | 43.54 | 6.75 | 3.87 | 13.29 |  | 23.16 | 10.47 | 39.34 | 5.04 | 0.00 | 24.07 | 1.42 | 0.85 | 2.58 |
| 18 | 10.48 | 0.00 | 33.78 | 13.55 | 0.00 | 31.63 | 3.80 | 2.24 | 7.26 |  | 27.18 | 12.88 | 48.40 | 7.29 | 0.00 | 28.40 | 2.14 | 1.20 | 4.42 |
| 19 | 10.33 | 0.00 | 42.51 | 17.82 | 0.00 | 41.95 | 4.07 | 2.08 | 9.75 |  | 27.33 | 13.49 | 44.72 | 4.68 | 0.00 | 23.40 | 1.89 | 1.09 | 3.73 |
| 20-69 | 21.09 | 11.96 | 36.59 | 12.67 | 0.00 | 24.50 | 2.39 | 1.86 | 3.13 |  | 25.41 | 16.06 | 31.18 | 0.78 | 0.00 | 10.92 | 2.43 | 1.98 | 3.04 |

LL: lower limit of confidence interval; UL: upper limit

Supplementary table 5. Proportion of the height variance explained by additive genetic, shared environmental and unique environmental with 95% confidence intervals by age, sex, parental education and cultural-geographic region.

|  | Additive genetic  variance | | | Shared environmental  variance | | | Unique environmental  variance | | |  | Additive genetic  variance | | | Shared environmental  variance | | | Unique environmental  variance | | |
| --- | --- | --- | --- | --- | --- | --- | --- | --- | --- | --- | --- | --- | --- | --- | --- | --- | --- | --- | --- |
| Age | a^2^ | LL | UL | c^2^ | LL | UL | e^2^ | LL | UL |  | a^2^ | LL | UL | c^2^ | LL | UL | e^2^ | LL | UL |
| European males with low parental education | | | | | | | | | |  | European females with low parental education | | | | | | | | |
| 1 | 0.45 | 0.38 | 0.53 | 0.43 | 0.35 | 0.50 | 0.12 | 0.11 | 0.13 |  | 0.52 | 0.44 | 0.62 | 0.34 | 0.25 | 0.42 | 0.13 | 0.12 | 0.15 |
| 2 | 0.57 | 0.48 | 0.67 | 0.32 | 0.22 | 0.41 | 0.11 | 0.09 | 0.12 |  | 0.60 | 0.50 | 0.70 | 0.30 | 0.20 | 0.39 | 0.10 | 0.09 | 0.12 |
| 3 | 0.69 | 0.60 | 0.81 | 0.21 | 0.09 | 0.30 | 0.10 | 0.09 | 0.12 |  | 0.76 | 0.67 | 0.87 | 0.16 | 0.04 | 0.24 | 0.08 | 0.07 | 0.09 |
| 4 | 0.32 | 0.14 | 0.56 | 0.53 | 0.29 | 0.70 | 0.15 | 0.12 | 0.20 |  | 0.71 | 0.48 | 0.91 | 0.18 | 0.00 | 0.41 | 0.11 | 0.09 | 0.15 |
| 5 | 0.67 | 0.53 | 0.83 | 0.27 | 0.10 | 0.40 | 0.07 | 0.05 | 0.08 |  | 0.64 | 0.51 | 0.80 | 0.30 | 0.14 | 0.43 | 0.06 | 0.05 | 0.08 |
| 6 | 0.65 | 0.33 | 0.92 | 0.23 | 0.00 | 0.54 | 0.12 | 0.08 | 0.19 |  | 0.93 | 0.89 | 0.95 | 0.00 | 0.00 | 0.34 | 0.07 | 0.05 | 0.11 |
| 7 | 0.80 | 0.66 | 0.96 | 0.16 | 0.00 | 0.30 | 0.05 | 0.04 | 0.06 |  | 0.69 | 0.56 | 0.84 | 0.24 | 0.08 | 0.37 | 0.07 | 0.06 | 0.09 |
| 8 | 0.60 | 0.46 | 0.78 | 0.35 | 0.17 | 0.49 | 0.06 | 0.04 | 0.07 |  | 0.60 | 0.45 | 0.81 | 0.33 | 0.13 | 0.48 | 0.07 | 0.05 | 0.08 |
| 9 | 0.56 | 0.37 | 0.83 | 0.34 | 0.07 | 0.54 | 0.09 | 0.07 | 0.12 |  | 0.47 | 0.33 | 0.66 | 0.48 | 0.29 | 0.62 | 0.05 | 0.04 | 0.07 |
| 10 | 0.81 | 0.66 | 0.95 | 0.14 | 0.00 | 0.29 | 0.05 | 0.04 | 0.06 |  | 0.71 | 0.56 | 0.88 | 0.21 | 0.03 | 0.35 | 0.09 | 0.07 | 0.11 |
| 11 | 0.72 | 0.55 | 0.93 | 0.21 | 0.00 | 0.37 | 0.07 | 0.06 | 0.09 |  | 0.79 | 0.61 | 0.93 | 0.13 | 0.00 | 0.31 | 0.08 | 0.07 | 0.10 |
| 12 | 0.86 | 0.69 | 0.94 | 0.07 | 0.00 | 0.24 | 0.07 | 0.06 | 0.08 |  | 0.91 | 0.89 | 0.92 | 0.00 | 0.00 | 0.16 | 0.09 | 0.08 | 0.11 |
| 13 | 0.67 | 0.48 | 0.94 | 0.29 | 0.02 | 0.49 | 0.03 | 0.02 | 0.04 |  | 0.65 | 0.49 | 0.86 | 0.29 | 0.08 | 0.46 | 0.05 | 0.04 | 0.07 |
| 14 | 0.88 | 0.71 | 0.93 | 0.04 | 0.00 | 0.21 | 0.08 | 0.07 | 0.10 |  | 0.79 | 0.62 | 0.89 | 0.08 | 0.00 | 0.25 | 0.13 | 0.11 | 0.15 |
| 15 | 0.86 | 0.58 | 0.92 | 0.03 | 0.00 | 0.31 | 0.11 | 0.08 | 0.15 |  | 0.71 | 0.48 | 0.92 | 0.20 | 0.00 | 0.43 | 0.10 | 0.07 | 0.13 |
| 16 | 0.52 | 0.34 | 0.76 | 0.39 | 0.15 | 0.57 | 0.09 | 0.07 | 0.12 |  | 0.67 | 0.47 | 0.91 | 0.22 | 0.00 | 0.42 | 0.11 | 0.08 | 0.14 |
| 17 | 0.75 | 0.54 | 0.90 | 0.13 | 0.00 | 0.34 | 0.12 | 0.10 | 0.16 |  | 0.59 | 0.42 | 0.80 | 0.28 | 0.07 | 0.44 | 0.14 | 0.11 | 0.17 |
| 18 | 0.86 | 0.64 | 0.96 | 0.09 | 0.00 | 0.32 | 0.05 | 0.03 | 0.06 |  | 0.74 | 0.53 | 0.90 | 0.14 | 0.00 | 0.35 | 0.12 | 0.10 | 0.16 |
| 19 | 0.78 | 0.46 | 0.92 | 0.11 | 0.00 | 0.43 | 0.11 | 0.07 | 0.16 |  | 0.67 | 0.41 | 0.89 | 0.19 | 0.00 | 0.45 | 0.14 | 0.10 | 0.19 |
| 20-69 | 0.71 | 0.58 | 0.85 | 0.17 | 0.03 | 0.30 | 0.12 | 0.10 | 0.14 |  | 0.80 | 0.68 | 0.91 | 0.11 | 0.00 | 0.23 | 0.09 | 0.08 | 0.11 |
| European males with intermediate parental education | | | | | | | | | |  | European females with intermediate parental education | | | | | | | | |
| 1 | 0.47 | 0.40 | 0.54 | 0.39 | 0.31 | 0.45 | 0.15 | 0.13 | 0.16 |  | 0.42 | 0.35 | 0.50 | 0.43 | 0.35 | 0.49 | 0.15 | 0.14 | 0.17 |
| 2 | 0.66 | 0.58 | 0.73 | 0.23 | 0.14 | 0.31 | 0.11 | 0.10 | 0.12 |  | 0.76 | 0.67 | 0.87 | 0.13 | 0.02 | 0.22 | 0.11 | 0.10 | 0.12 |
| 3 | 0.68 | 0.59 | 0.78 | 0.24 | 0.14 | 0.32 | 0.08 | 0.07 | 0.09 |  | 0.65 | 0.57 | 0.75 | 0.25 | 0.15 | 0.33 | 0.10 | 0.09 | 0.11 |
| 4 | 0.62 | 0.42 | 0.89 | 0.32 | 0.05 | 0.52 | 0.07 | 0.05 | 0.10 |  | 0.82 | 0.54 | 0.95 | 0.11 | 0.00 | 0.39 | 0.07 | 0.05 | 0.10 |
| 5 | 0.74 | 0.62 | 0.78 | 0.21 | 0.08 | 0.32 | 0.05 | 0.05 | 0.06 |  | 0.65 | 0.54 | 0.78 | 0.29 | 0.16 | 0.39 | 0.07 | 0.06 | 0.08 |
| 6 | 0.80 | 0.47 | 0.96 | 0.13 | 0.00 | 0.46 | 0.06 | 0.04 | 0.12 |  | 0.84 | 0.52 | 0.97 | 0.11 | 0.00 | 0.44 | 0.04 | 0.03 | 0.07 |
| 7 | 0.62 | 0.53 | 0.72 | 0.33 | 0.22 | 0.42 | 0.05 | 0.05 | 0.06 |  | 0.69 | 0.58 | 0.81 | 0.23 | 0.11 | 0.34 | 0.08 | 0.07 | 0.09 |
| 8 | 0.77 | 0.62 | 0.95 | 0.18 | 0.00 | 0.33 | 0.05 | 0.04 | 0.06 |  | 0.86 | 0.70 | 0.95 | 0.08 | 0.00 | 0.24 | 0.05 | 0.04 | 0.07 |
| 9 | 0.50 | 0.34 | 0.73 | 0.44 | 0.21 | 0.60 | 0.06 | 0.05 | 0.08 |  | 0.70 | 0.48 | 0.96 | 0.25 | 0.00 | 0.47 | 0.05 | 0.03 | 0.06 |
| 10 | 0.78 | 0.66 | 0.91 | 0.16 | 0.03 | 0.28 | 0.06 | 0.05 | 0.07 |  | 0.67 | 0.57 | 0.80 | 0.25 | 0.12 | 0.36 | 0.08 | 0.07 | 0.09 |
| 11 | 0.83 | 0.63 | 0.95 | 0.10 | 0.00 | 0.31 | 0.06 | 0.05 | 0.08 |  | 0.74 | 0.56 | 0.95 | 0.20 | 0.00 | 0.39 | 0.05 | 0.04 | 0.07 |
| 12 | 0.86 | 0.73 | 0.94 | 0.08 | 0.00 | 0.21 | 0.06 | 0.06 | 0.08 |  | 0.80 | 0.67 | 0.93 | 0.12 | 0.00 | 0.25 | 0.08 | 0.07 | 0.09 |
| 13 | 0.67 | 0.50 | 0.89 | 0.25 | 0.04 | 0.42 | 0.07 | 0.06 | 0.10 |  | 0.95 | 0.80 | 0.96 | 0.00 | 0.00 | 0.16 | 0.05 | 0.04 | 0.06 |
| 14 | 0.82 | 0.63 | 0.93 | 0.09 | 0.00 | 0.28 | 0.09 | 0.07 | 0.11 |  | 0.81 | 0.64 | 0.93 | 0.10 | 0.00 | 0.27 | 0.09 | 0.07 | 0.11 |
| 15 | 0.68 | 0.46 | 0.90 | 0.20 | 0.00 | 0.41 | 0.12 | 0.09 | 0.16 |  | 0.74 | 0.53 | 0.92 | 0.17 | 0.00 | 0.37 | 0.09 | 0.07 | 0.12 |
| 16 | 0.81 | 0.62 | 0.91 | 0.08 | 0.00 | 0.26 | 0.11 | 0.09 | 0.14 |  | 0.85 | 0.69 | 0.92 | 0.05 | 0.00 | 0.21 | 0.09 | 0.08 | 0.11 |
| 17 | 0.80 | 0.64 | 0.91 | 0.09 | 0.00 | 0.25 | 0.11 | 0.09 | 0.13 |  | 0.75 | 0.61 | 0.90 | 0.14 | 0.00 | 0.29 | 0.11 | 0.09 | 0.13 |
| 18 | 0.73 | 0.49 | 0.88 | 0.12 | 0.00 | 0.35 | 0.15 | 0.11 | 0.21 |  | 0.85 | 0.63 | 0.92 | 0.05 | 0.00 | 0.27 | 0.10 | 0.08 | 0.13 |
| 19 | 0.86 | 0.58 | 0.90 | 0.01 | 0.00 | 0.28 | 0.13 | 0.10 | 0.18 |  | 0.88 | 0.65 | 0.91 | 0.00 | 0.00 | 0.24 | 0.11 | 0.09 | 0.15 |
| 20-69 | 0.80 | 0.70 | 0.91 | 0.12 | 0.00 | 0.22 | 0.08 | 0.07 | 0.09 |  | 0.80 | 0.69 | 0.87 | 0.06 | 0.00 | 0.16 | 0.14 | 0.13 | 0.16 |
| European males with high parental education | | | | | | | | | |  | European females with high parental education | | | | | | | | |
| 1 | 0.54 | 0.44 | 0.66 | 0.31 | 0.20 | 0.40 | 0.15 | 0.13 | 0.17 |  | 0.53 | 0.43 | 0.64 | 0.31 | 0.20 | 0.41 | 0.16 | 0.14 | 0.18 |
| 2 | 0.69 | 0.58 | 0.82 | 0.19 | 0.11 | 0.30 | 0.11 | 0.10 | 0.13 |  | 0.58 | 0.48 | 0.69 | 0.32 | 0.21 | 0.42 | 0.10 | 0.09 | 0.12 |
| 3 | 0.68 | 0.57 | 0.81 | 0.22 | 0.09 | 0.33 | 0.10 | 0.09 | 0.12 |  | 0.63 | 0.52 | 0.76 | 0.27 | 0.14 | 0.37 | 0.10 | 0.09 | 0.12 |
| 4 | 0.66 | 0.45 | 0.91 | 0.24 | 0.00 | 0.44 | 0.10 | 0.08 | 0.14 |  | 0.75 | 0.55 | 0.94 | 0.17 | 0.00 | 0.37 | 0.08 | 0.06 | 0.10 |
| 5 | 0.75 | 0.63 | 0.89 | 0.20 | 0.05 | 0.32 | 0.06 | 0.05 | 0.07 |  | 0.73 | 0.61 | 0.78 | 0.19 | 0.04 | 0.31 | 0.08 | 0.07 | 0.10 |
| 6 | 0.72 | 0.46 | 0.95 | 0.21 | 0.00 | 0.47 | 0.07 | 0.04 | 0.12 |  | 0.70 | 0.41 | 0.94 | 0.22 | 0.00 | 0.51 | 0.08 | 0.05 | 0.13 |
| 7 | 0.66 | 0.54 | 0.80 | 0.29 | 0.14 | 0.41 | 0.05 | 0.04 | 0.06 |  | 0.78 | 0.63 | 0.94 | 0.16 | 0.00 | 0.30 | 0.06 | 0.05 | 0.08 |
| 8 | 0.73 | 0.55 | 0.92 | 0.18 | 0.00 | 0.37 | 0.09 | 0.07 | 0.11 |  | 0.85 | 0.65 | 0.95 | 0.09 | 0.00 | 0.29 | 0.06 | 0.05 | 0.07 |
| 9 | 0.88 | 0.62 | 0.96 | 0.07 | 0.00 | 0.33 | 0.05 | 0.04 | 0.07 |  | 0.91 | 0.88 | 0.93 | 0.00 | 0.00 | 0.31 | 0.09 | 0.07 | 0.12 |
| 10 | 0.71 | 0.59 | 0.86 | 0.24 | 0.09 | 0.36 | 0.05 | 0.04 | 0.06 |  | 0.62 | 0.50 | 0.78 | 0.32 | 0.16 | 0.44 | 0.06 | 0.05 | 0.07 |
| 11 | 0.67 | 0.48 | 0.90 | 0.23 | 0.00 | 0.42 | 0.10 | 0.08 | 0.13 |  | 0.92 | 0.71 | 0.94 | 0.00 | 0.00 | 0.21 | 0.08 | 0.06 | 0.10 |
| 12 | 0.76 | 0.62 | 0.92 | 0.16 | 0.00 | 0.30 | 0.07 | 0.06 | 0.09 |  | 0.83 | 0.68 | 0.94 | 0.10 | 0.00 | 0.25 | 0.07 | 0.06 | 0.08 |
| 13 | 0.65 | 0.46 | 0.92 | 0.27 | 0.00 | 0.46 | 0.08 | 0.06 | 0.11 |  | 0.58 | 0.39 | 0.85 | 0.33 | 0.07 | 0.52 | 0.08 | 0.06 | 0.11 |
| 14 | 0.78 | 0.60 | 0.91 | 0.11 | 0.00 | 0.28 | 0.11 | 0.09 | 0.14 |  | 0.69 | 0.53 | 0.87 | 0.17 | 0.00 | 0.33 | 0.14 | 0.11 | 0.16 |
| 15 | 0.49 | 0.28 | 0.79 | 0.39 | 0.10 | 0.59 | 0.12 | 0.09 | 0.16 |  | 0.87 | 0.60 | 0.93 | 0.04 | 0.00 | 0.31 | 0.09 | 0.07 | 0.12 |
| 16 | 0.62 | 0.40 | 0.89 | 0.27 | 0.00 | 0.47 | 0.12 | 0.09 | 0.16 |  | 0.65 | 0.43 | 0.90 | 0.24 | 0.00 | 0.45 | 0.11 | 0.09 | 0.15 |
| 17 | 0.89 | 0.69 | 0.94 | 0.03 | 0.00 | 0.23 | 0.08 | 0.06 | 0.10 |  | 0.65 | 0.44 | 0.84 | 0.17 | 0.00 | 0.37 | 0.18 | 0.15 | 0.23 |
| 18 | 0.74 | 0.50 | 0.92 | 0.15 | 0.00 | 0.39 | 0.11 | 0.08 | 0.15 |  | 0.73 | 0.48 | 0.88 | 0.12 | 0.00 | 0.35 | 0.16 | 0.12 | 0.20 |
| 19 | 0.88 | 0.81 | 0.92 | 0.00 | 0.00 | 0.32 | 0.12 | 0.08 | 0.19 |  | 0.84 | 0.55 | 0.92 | 0.05 | 0.00 | 0.33 | 0.11 | 0.08 | 0.16 |
| 20-69 | 0.73 | 0.59 | 0.91 | 0.17 | 0.00 | 0.32 | 0.09 | 0.08 | 0.12 |  | 0.73 | 0.59 | 0.88 | 0.17 | 0.02 | 0.30 | 0.11 | 0.09 | 0.13 |
| North-American and Australian males with low parental education | | | | | | | | | |  | North-American and Australian females with low parental education | | | | | | | | |
| 1 |  |  |  |  |  |  |  |  |  |  |  |  |  |  |  |  |  |  |  |
| 2 | 0.95 | 0.84 | 0.98 | 0.00 | 0.00 | 0.21 | 0.05 | 0.02 | 0.16 |  | 0.39 | 0.00 | 0.91 | 0.46 | 0.00 | 0.84 | 0.16 | 0.07 | 0.37 |
| 3 | 0.43 | 0.23 | 0.75 | 0.49 | 0.17 | 0.69 | 0.07 | 0.05 | 0.12 |  | 0.26 | 0.04 | 0.63 | 0.49 | 0.14 | 0.70 | 0.25 | 0.17 | 0.37 |
| 4 | 0.36 | 0.24 | 0.51 | 0.60 | 0.44 | 0.71 | 0.05 | 0.03 | 0.07 |  | 0.31 | 0.13 | 0.54 | 0.55 | 0.33 | 0.71 | 0.13 | 0.10 | 0.19 |
| 5 | 0.85 | 0.58 | 0.97 | 0.12 | 0.00 | 0.39 | 0.03 | 0.02 | 0.05 |  | 0.42 | 0.23 | 0.75 | 0.50 | 0.17 | 0.68 | 0.08 | 0.05 | 0.14 |
| 6 | 0.23 | 0.10 | 0.45 | 0.71 | 0.49 | 0.83 | 0.06 | 0.04 | 0.10 |  | 0.80 | 0.50 | 0.93 | 0.13 | 0.01 | 0.42 | 0.07 | 0.04 | 0.13 |
| 7 | 0.29 | 0.14 | 0.55 | 0.66 | 0.41 | 0.81 | 0.04 | 0.03 | 0.08 |  | 0.38 | 0.18 | 0.69 | 0.56 | 0.25 | 0.74 | 0.07 | 0.04 | 0.11 |
| 8 | 0.24 | 0.08 | 0.52 | 0.69 | 0.42 | 0.84 | 0.06 | 0.04 | 0.12 |  | 0.82 | 0.45 | 0.96 | 0.12 | 0.00 | 0.49 | 0.06 | 0.03 | 0.12 |
| 9 | 0.41 | 0.24 | 0.68 | 0.51 | 0.24 | 0.68 | 0.07 | 0.05 | 0.11 |  | 0.49 | 0.25 | 0.92 | 0.43 | 0.00 | 0.66 | 0.08 | 0.05 | 0.12 |
| 10 | 0.92 | 0.56 | 0.96 | 0.01 | 0.00 | 0.37 | 0.07 | 0.04 | 0.12 |  | 0.68 | 0.41 | 0.96 | 0.27 | 0.00 | 0.54 | 0.05 | 0.03 | 0.08 |
| 11 | 0.37 | 0.01 | 0.84 | 0.43 | 0.00 | 0.73 | 0.19 | 0.12 | 0.31 |  | 0.76 | 0.46 | 0.96 | 0.18 | 0.00 | 0.49 | 0.06 | 0.04 | 0.10 |
| 12 | 0.55 | 0.28 | 0.90 | 0.33 | 0.00 | 0.59 | 0.12 | 0.08 | 0.19 |  | 0.41 | 0.13 | 0.82 | 0.44 | 0.04 | 0.69 | 0.15 | 0.10 | 0.23 |
| 13 | 0.82 | 0.44 | 0.89 | 0.00 | 0.00 | 0.36 | 0.18 | 0.11 | 0.28 |  | 0.83 | 0.72 | 0.89 | 0.00 | 0.00 | 0.41 | 0.17 | 0.11 | 0.28 |
| 14 | 0.91 | 0.86 | 0.94 | 0.00 | 0.00 | 0.26 | 0.09 | 0.06 | 0.14 |  | 0.62 | 0.29 | 0.89 | 0.22 | 0.00 | 0.53 | 0.16 | 0.10 | 0.25 |
| 15 | 0.80 | 0.51 | 0.97 | 0.15 | 0.00 | 0.44 | 0.05 | 0.03 | 0.08 |  | 0.94 | 0.90 | 0.97 | 0.00 | 0.00 | 0.29 | 0.06 | 0.03 | 0.10 |
| 16 | 0.47 | 0.17 | 0.86 | 0.36 | 0.00 | 0.63 | 0.17 | 0.11 | 0.26 |  | 0.57 | 0.33 | 0.90 | 0.33 | 0.00 | 0.56 | 0.10 | 0.07 | 0.15 |
| 17 | 0.81 | 0.53 | 0.93 | 0.09 | 0.00 | 0.37 | 0.10 | 0.07 | 0.14 |  | 0.70 | 0.44 | 0.95 | 0.23 | 0.00 | 0.50 | 0.07 | 0.05 | 0.10 |
| 18 | 0.70 | 0.50 | 0.93 | 0.22 | 0.00 | 0.42 | 0.08 | 0.06 | 0.11 |  | 0.50 | 0.24 | 0.94 | 0.43 | 0.00 | 0.69 | 0.07 | 0.04 | 0.11 |
| 19 | 0.65 | 0.37 | 0.93 | 0.26 | 0.00 | 0.53 | 0.09 | 0.06 | 0.15 |  | 0.92 | 0.63 | 0.94 | 0.00 | 0.00 | 0.29 | 0.08 | 0.06 | 0.13 |
| 20-69 | 0.71 | 0.63 | 0.79 | 0.16 | 0.08 | 0.24 | 0.13 | 0.12 | 0.14 |  | 0.65 | 0.58 | 0.72 | 0.21 | 0.14 | 0.28 | 0.14 | 0.13 | 0.15 |
| North-American and Australian males with intermediate parental education | | | | | | | | | |  | North-American and Australian females with intermediate parental education | | | | | | | | |
| 1 |  |  |  |  |  |  |  |  |  |  |  |  |  |  |  |  |  |  |  |
| 2 | 0.31 | 0.00 | 0.76 | 0.36 | 0.00 | 0.67 | 0.33 | 0.21 | 0.53 |  | 0.60 | 0.33 | 0.95 | 0.34 | 0.00 | 0.60 | 0.07 | 0.04 | 0.12 |
| 3 | 0.59 | 0.40 | 0.86 | 0.33 | 0.06 | 0.52 | 0.08 | 0.05 | 0.12 |  | 0.47 | 0.31 | 0.69 | 0.49 | 0.26 | 0.65 | 0.04 | 0.03 | 0.06 |
| 4 | 0.45 | 0.30 | 0.66 | 0.52 | 0.30 | 0.66 | 0.04 | 0.02 | 0.06 |  | 0.55 | 0.35 | 0.85 | 0.38 | 0.08 | 0.58 | 0.07 | 0.05 | 0.11 |
| 5 | 0.42 | 0.27 | 0.69 | 0.56 | 0.30 | 0.72 | 0.01 | 0.01 | 0.03 |  | 0.40 | 0.12 | 0.84 | 0.50 | 0.06 | 0.74 | 0.10 | 0.06 | 0.20 |
| 6 | 0.29 | 0.12 | 0.65 | 0.66 | 0.31 | 0.83 | 0.04 | 0.02 | 0.09 |  | 0.43 | 0.22 | 0.76 | 0.51 | 0.18 | 0.71 | 0.06 | 0.03 | 0.11 |
| 7 | 0.82 | 0.48 | 0.97 | 0.12 | 0.00 | 0.45 | 0.06 | 0.03 | 0.13 |  | 0.57 | 0.34 | 0.92 | 0.40 | 0.05 | 0.63 | 0.03 | 0.02 | 0.06 |
| 8 | 0.86 | 0.49 | 0.97 | 0.08 | 0.00 | 0.45 | 0.06 | 0.03 | 0.13 |  | 0.20 | 0.01 | 0.72 | 0.54 | 0.05 | 0.76 | 0.26 | 0.15 | 0.43 |
| 9 | 0.75 | 0.45 | 0.89 | 0.09 | 0.00 | 0.37 | 0.16 | 0.11 | 0.24 |  | 0.85 | 0.55 | 0.96 | 0.09 | 0.00 | 0.39 | 0.06 | 0.04 | 0.09 |
| 10 | 0.94 | 0.90 | 0.97 | 0.00 | 0.00 | 0.19 | 0.06 | 0.03 | 0.10 |  | 0.44 | 0.20 | 0.83 | 0.48 | 0.09 | 0.70 | 0.08 | 0.05 | 0.15 |
| 11 | 0.62 | 0.31 | 0.95 | 0.31 | 0.00 | 0.61 | 0.08 | 0.04 | 0.14 |  | 0.81 | 0.42 | 0.97 | 0.15 | 0.00 | 0.54 | 0.04 | 0.03 | 0.08 |
| 12 | 0.74 | 0.40 | 0.94 | 0.16 | 0.00 | 0.50 | 0.10 | 0.06 | 0.16 |  | 0.58 | 0.10 | 0.80 | 0.12 | 0.00 | 0.53 | 0.30 | 0.20 | 0.45 |
| 13 | 0.30 | 0.07 | 0.66 | 0.59 | 0.24 | 0.79 | 0.11 | 0.07 | 0.19 |  | 0.68 | 0.29 | 0.90 | 0.16 | 0.00 | 0.53 | 0.16 | 0.10 | 0.25 |
| 14 | 0.88 | 0.80 | 0.93 | 0.00 | 0.00 | 0.47 | 0.12 | 0.07 | 0.20 |  | 0.28 | 0.00 | 0.78 | 0.44 | 0.00 | 0.73 | 0.28 | 0.18 | 0.44 |
| 15 | 0.93 | 0.87 | 0.95 | 0.00 | 0.00 | 0.27 | 0.07 | 0.05 | 0.13 |  | 0.86 | 0.44 | 0.92 | 0.02 | 0.00 | 0.43 | 0.12 | 0.08 | 0.20 |
| 16 | 0.61 | 0.38 | 0.96 | 0.34 | 0.00 | 0.57 | 0.04 | 0.03 | 0.07 |  | 0.91 | 0.74 | 0.94 | 0.00 | 0.00 | 0.17 | 0.09 | 0.06 | 0.13 |
| 17 | 0.85 | 0.53 | 0.95 | 0.07 | 0.00 | 0.40 | 0.08 | 0.05 | 0.11 |  | 0.66 | 0.41 | 0.92 | 0.24 | 0.00 | 0.49 | 0.10 | 0.07 | 0.14 |
| 18 | 0.88 | 0.75 | 0.92 | 0.00 | 0.00 | 0.13 | 0.12 | 0.08 | 0.17 |  | 0.54 | 0.10 | 0.82 | 0.19 | 0.00 | 0.55 | 0.27 | 0.18 | 0.42 |
| 19 | 0.56 | 0.30 | 0.96 | 0.39 | 0.00 | 0.65 | 0.05 | 0.03 | 0.07 |  | 0.21 | 0.00 | 0.71 | 0.56 | 0.08 | 0.80 | 0.23 | 0.14 | 0.37 |
| 20-69 | 0.63 | 0.56 | 0.71 | 0.24 | 0.17 | 0.31 | 0.12 | 0.11 | 0.13 |  | 0.69 | 0.62 | 0.76 | 0.19 | 0.12 | 0.26 | 0.12 | 0.11 | 0.13 |
| North-American and Australian males with high parental education | | | | | | | | | |  | North-American and Australian females with high parental education | | | | | | | | |
| 1 |  |  |  |  |  |  |  |  |  |  |  |  |  |  |  |  |  |  |  |
| 2 | 0.25 | 0.00 | 0.81 | 0.63 | 0.07 | 0.88 | 0.12 | 0.05 | 0.28 |  | 0.94 | 0.85 | 0.97 | 0.00 | 0.00 | 0.50 | 0.06 | 0.03 | 0.15 |
| 3 | 0.41 | 0.24 | 0.67 | 0.54 | 0.28 | 0.70 | 0.05 | 0.03 | 0.09 |  | 0.73 | 0.46 | 0.96 | 0.22 | 0.00 | 0.49 | 0.05 | 0.03 | 0.08 |
| 4 | 0.72 | 0.48 | 0.93 | 0.19 | 0.00 | 0.43 | 0.09 | 0.06 | 0.14 |  | 0.44 | 0.28 | 0.67 | 0.51 | 0.28 | 0.67 | 0.05 | 0.03 | 0.08 |
| 5 | 0.67 | 0.41 | 0.97 | 0.29 | 0.00 | 0.55 | 0.04 | 0.02 | 0.07 |  | 0.45 | 0.24 | 0.78 | 0.51 | 0.18 | 0.71 | 0.04 | 0.02 | 0.09 |
| 6 | 0.33 | 0.11 | 0.68 | 0.56 | 0.22 | 0.76 | 0.11 | 0.07 | 0.19 |  | 0.88 | 0.53 | 0.98 | 0.08 | 0.00 | 0.43 | 0.04 | 0.02 | 0.08 |
| 7 | 0.42 | 0.23 | 0.74 | 0.55 | 0.22 | 0.73 | 0.03 | 0.02 | 0.06 |  | 0.70 | 0.40 | 0.96 | 0.23 | 0.00 | 0.53 | 0.07 | 0.04 | 0.13 |
| 8 | 0.59 | 0.30 | 0.97 | 0.37 | 0.00 | 0.66 | 0.04 | 0.02 | 0.08 |  | 0.48 | 0.21 | 0.94 | 0.44 | 0.00 | 0.70 | 0.07 | 0.04 | 0.15 |
| 9 | 0.78 | 0.53 | 0.96 | 0.16 | 0.00 | 0.41 | 0.05 | 0.04 | 0.08 |  | 0.50 | 0.33 | 0.75 | 0.43 | 0.19 | 0.60 | 0.07 | 0.05 | 0.10 |
| 10 | 0.82 | 0.51 | 0.96 | 0.11 | 0.00 | 0.42 | 0.07 | 0.04 | 0.12 |  | 0.46 | 0.25 | 0.88 | 0.50 | 0.08 | 0.72 | 0.04 | 0.02 | 0.07 |
| 11 | 0.34 | 0.00 | 0.79 | 0.33 | 0.00 | 0.69 | 0.33 | 0.19 | 0.58 |  | 0.83 | 0.48 | 0.96 | 0.10 | 0.00 | 0.45 | 0.07 | 0.04 | 0.12 |
| 12 | 0.81 | 0.69 | 0.88 | 0.00 | 0.00 | 0.40 | 0.19 | 0.12 | 0.31 |  | 0.83 | 0.54 | 0.97 | 0.12 | 0.00 | 0.40 | 0.05 | 0.03 | 0.09 |
| 13 | 0.73 | 0.42 | 0.95 | 0.19 | 0.00 | 0.49 | 0.08 | 0.05 | 0.14 |  | 0.93 | 0.88 | 0.96 | 0.00 | 0.00 | 0.38 | 0.07 | 0.04 | 0.12 |
| 14 | 0.89 | 0.52 | 0.96 | 0.04 | 0.00 | 0.41 | 0.07 | 0.04 | 0.11 |  | 0.62 | 0.34 | 0.95 | 0.31 | 0.00 | 0.60 | 0.07 | 0.04 | 0.11 |
| 15 | 0.72 | 0.42 | 0.96 | 0.22 | 0.00 | 0.53 | 0.06 | 0.04 | 0.09 |  | 0.53 | 0.15 | 0.90 | 0.31 | 0.00 | 0.64 | 0.16 | 0.09 | 0.29 |
| 16 | 0.93 | 0.88 | 0.95 | 0.00 | 0.00 | 0.19 | 0.07 | 0.05 | 0.12 |  | 0.64 | 0.30 | 0.89 | 0.20 | 0.00 | 0.50 | 0.16 | 0.11 | 0.26 |
| 17 | 0.60 | 0.39 | 0.90 | 0.33 | 0.03 | 0.53 | 0.07 | 0.05 | 0.11 |  | 0.55 | 0.32 | 0.89 | 0.37 | 0.02 | 0.59 | 0.08 | 0.06 | 0.13 |
| 18 | 0.70 | 0.50 | 0.93 | 0.22 | 0.00 | 0.42 | 0.08 | 0.06 | 0.11 |  | 0.75 | 0.47 | 0.97 | 0.21 | 0.00 | 0.49 | 0.04 | 0.03 | 0.07 |
| 19 | 0.52 | 0.15 | 0.88 | 0.31 | 0.00 | 0.66 | 0.17 | 0.11 | 0.26 |  | 0.64 | 0.29 | 0.90 | 0.21 | 0.00 | 0.54 | 0.15 | 0.10 | 0.24 |
| 20-69 | 0.47 | 0.40 | 0.54 | 0.36 | 0.29 | 0.43 | 0.17 | 0.16 | 0.19 |  | 0.56 | 0.49 | 0.64 | 0.29 | 0.21 | 0.35 | 0.15 | 0.14 | 0.16 |
|  |  |  |  |  |  |  |  |  |  |  |  |  |  |  |  |  |  |  |  |
| East-Asian males with low parental education | | | | | | | | | |  | East-Asian females with low parental education | | | | | | | | |
| 1 | 0.29 | 0.01 | 0.77 | 0.61 | 0.13 | 0.85 | 0.10 | 0.06 | 0.19 |  | 0.15 | 0.00 | 0.61 | 0.77 | 0.31 | 0.92 | 0.08 | 0.05 | 0.15 |
| 2 | 0.67 | 0.13 | 0.89 | 0.13 | 0.00 | 0.60 | 0.20 | 0.10 | 0.40 |  | 0.40 | 0.08 | 0.93 | 0.49 | 0.00 | 0.79 | 0.10 | 0.05 | 0.23 |
| 3 | 0.85 | 0.32 | 0.96 | 0.08 | 0.00 | 0.62 | 0.06 | 0.04 | 0.11 |  | 0.55 | 0.14 | 0.87 | 0.25 | 0.00 | 0.62 | 0.21 | 0.12 | 0.35 |
| 4 | 0.06 | 0.00 | 0.50 | 0.73 | 0.30 | 0.87 | 0.22 | 0.10 | 0.42 |  | 0.06 | 0.00 | 0.53 | 0.88 | 0.41 | 0.97 | 0.05 | 0.02 | 0.12 |
| 5 | 0.53 | 0.16 | 0.93 | 0.36 | 0.00 | 0.70 | 0.11 | 0.06 | 0.22 |  | 0.65 | 0.26 | 0.94 | 0.26 | 0.00 | 0.64 | 0.09 | 0.05 | 0.18 |
| 6 | 0.21 | 0.00 | 0.72 | 0.66 | 0.16 | 0.86 | 0.13 | 0.07 | 0.27 |  | 0.21 | 0.02 | 0.95 | 0.74 | 0.00 | 0.92 | 0.05 | 0.03 | 0.11 |
| 7 | 0.81 | 0.31 | 0.89 | 0.01 | 0.00 | 0.49 | 0.18 | 0.11 | 0.29 |  | 0.68 | 0.33 | 0.97 | 0.26 | 0.00 | 0.62 | 0.05 | 0.03 | 0.10 |
| 8 | 0.37 | 0.17 | 0.78 | 0.60 | 0.19 | 0.80 | 0.03 | 0.02 | 0.07 |  | 0.57 | 0.22 | 0.98 | 0.39 | 0.00 | 0.75 | 0.04 | 0.02 | 0.08 |
| 9 | 0.37 | 0.10 | 0.82 | 0.52 | 0.08 | 0.77 | 0.11 | 0.06 | 0.19 |  | 0.36 | 0.15 | 0.74 | 0.58 | 0.20 | 0.78 | 0.06 | 0.03 | 0.11 |
| 10 | 0.96 | 0.56 | 0.99 | 0.03 | 0.00 | 0.42 | 0.02 | 0.01 | 0.03 |  | 0.96 | 0.92 | 0.98 | 0.00 | 0.00 | 0.43 | 0.04 | 0.02 | 0.08 |
| 11 | 0.31 | 0.13 | 0.63 | 0.61 | 0.30 | 0.79 | 0.07 | 0.04 | 0.12 |  | 0.94 | 0.91 | 0.96 | 0.00 | 0.00 | 0.31 | 0.06 | 0.04 | 0.09 |
| 12 | 0.12 | 0.00 | 0.48 | 0.79 | 0.43 | 0.92 | 0.09 | 0.05 | 0.15 |  | 0.41 | 0.13 | 0.92 | 0.49 | 0.00 | 0.76 | 0.10 | 0.06 | 0.18 |
| 13 | 0.56 | 0.23 | 0.97 | 0.38 | 0.00 | 0.71 | 0.05 | 0.03 | 0.10 |  | 0.57 | 0.19 | 0.92 | 0.31 | 0.00 | 0.66 | 0.13 | 0.08 | 0.22 |
| 14 | 0.00 | 0.00 | 0.17 | 0.87 | 0.79 | 0.93 | 0.13 | 0.07 | 0.21 |  | 0.15 | 0.00 | 0.64 | 0.62 | 0.16 | 0.84 | 0.23 | 0.11 | 0.47 |
| 15 | 0.20 | 0.00 | 0.70 | 0.74 | 0.24 | 0.92 | 0.06 | 0.03 | 0.13 |  | 0.68 | 0.07 | 0.88 | 0.12 | 0.00 | 0.71 | 0.20 | 0.12 | 0.34 |
| 16 | 0.08 | 0.00 | 0.54 | 0.85 | 0.38 | 0.96 | 0.07 | 0.04 | 0.15 |  | 0.69 | 0.23 | 0.99 | 0.28 | 0.00 | 0.75 | 0.03 | 0.01 | 0.06 |
| 17 | 0.19 | 0.00 | 0.87 | 0.68 | 0.00 | 0.89 | 0.14 | 0.08 | 0.25 |  | 0.24 | 0.00 | 0.76 | 0.40 | 0.00 | 0.73 | 0.36 | 0.22 | 0.58 |
| 18 | 0.41 | 0.15 | 0.97 | 0.54 | 0.00 | 0.81 | 0.04 | 0.02 | 0.09 |  | 0.39 | 0.09 | 0.94 | 0.52 | 0.00 | 0.81 | 0.08 | 0.05 | 0.16 |
| 19 | 0.01 | 0.00 | 0.25 | 0.92 | 0.67 | 0.95 | 0.07 | 0.04 | 0.13 |  | 0.76 | 0.34 | 0.95 | 0.16 | 0.00 | 0.59 | 0.08 | 0.04 | 0.15 |
| 20-69 | 0.80 | 0.44 | 0.95 | 0.12 | 0.00 | 0.49 | 0.08 | 0.05 | 0.11 |  | 0.51 | 0.30 | 0.88 | 0.41 | 0.05 | 0.63 | 0.07 | 0.06 | 0.10 |
| East-Asian males with intermediate parental education | | | | | | | | | |  | East-Asian females with intermediate parental education | | | | | | | | |
| 1 | 0.38 | 0.16 | 0.76 | 0.53 | 0.14 | 0.73 | 0.10 | 0.07 | 0.15 |  | 0.31 | 0.02 | 0.70 | 0.43 | 0.06 | 0.69 | 0.25 | 0.18 | 0.36 |
| 2 | 0.67 | 0.36 | 0.95 | 0.28 | 0.00 | 0.58 | 0.06 | 0.04 | 0.09 |  | 0.28 | 0.12 | 0.61 | 0.66 | 0.33 | 0.83 | 0.05 | 0.04 | 0.08 |
| 3 | 0.91 | 0.58 | 0.96 | 0.02 | 0.00 | 0.35 | 0.06 | 0.04 | 0.10 |  | 0.40 | 0.19 | 0.71 | 0.50 | 0.19 | 0.69 | 0.10 | 0.07 | 0.15 |
| 4 | 0.32 | 0.14 | 0.65 | 0.63 | 0.29 | 0.80 | 0.05 | 0.03 | 0.09 |  | 0.37 | 0.19 | 0.68 | 0.58 | 0.27 | 0.76 | 0.05 | 0.03 | 0.08 |
| 5 | 0.11 | 0.00 | 0.35 | 0.79 | 0.55 | 0.90 | 0.10 | 0.06 | 0.18 |  | 0.84 | 0.47 | 0.97 | 0.12 | 0.00 | 0.49 | 0.04 | 0.02 | 0.07 |
| 6 | 0.52 | 0.31 | 0.86 | 0.43 | 0.09 | 0.65 | 0.05 | 0.03 | 0.08 |  | 0.38 | 0.22 | 0.66 | 0.58 | 0.30 | 0.74 | 0.04 | 0.03 | 0.06 |
| 7 | 0.59 | 0.29 | 0.94 | 0.32 | 0.00 | 0.62 | 0.09 | 0.05 | 0.15 |  | 0.56 | 0.30 | 0.96 | 0.39 | 0.00 | 0.65 | 0.05 | 0.03 | 0.08 |
| 8 | 0.17 | 0.00 | 0.45 | 0.72 | 0.44 | 0.87 | 0.11 | 0.07 | 0.20 |  | 0.61 | 0.36 | 0.94 | 0.33 | 0.00 | 0.58 | 0.06 | 0.04 | 0.10 |
| 9 | 0.58 | 0.31 | 0.94 | 0.34 | 0.00 | 0.61 | 0.08 | 0.04 | 0.14 |  | 0.90 | 0.85 | 0.94 | 0.00 | 0.00 | 0.33 | 0.10 | 0.06 | 0.15 |
| 10 | 0.45 | 0.22 | 0.87 | 0.47 | 0.05 | 0.71 | 0.07 | 0.04 | 0.12 |  | 0.63 | 0.37 | 0.93 | 0.29 | 0.00 | 0.54 | 0.08 | 0.06 | 0.12 |
| 11 | 0.44 | 0.16 | 0.85 | 0.44 | 0.04 | 0.69 | 0.12 | 0.07 | 0.21 |  | 0.68 | 0.39 | 0.98 | 0.29 | 0.00 | 0.59 | 0.03 | 0.02 | 0.04 |
| 12 | 0.95 | 0.55 | 0.97 | 0.00 | 0.00 | 0.41 | 0.05 | 0.03 | 0.08 |  | 0.75 | 0.43 | 0.95 | 0.17 | 0.00 | 0.50 | 0.07 | 0.05 | 0.12 |
| 13 | 0.14 | 0.00 | 0.52 | 0.63 | 0.25 | 0.81 | 0.23 | 0.13 | 0.39 |  | 0.30 | 0.09 | 0.68 | 0.60 | 0.23 | 0.81 | 0.09 | 0.06 | 0.15 |
| 14 | 0.66 | 0.29 | 0.96 | 0.26 | 0.00 | 0.63 | 0.07 | 0.04 | 0.13 |  | 0.91 | 0.51 | 0.94 | 0.00 | 0.00 | 0.40 | 0.09 | 0.06 | 0.15 |
| 15 | 0.42 | 0.00 | 0.83 | 0.29 | 0.00 | 0.73 | 0.29 | 0.16 | 0.55 |  | 0.54 | 0.15 | 0.90 | 0.32 | 0.00 | 0.68 | 0.14 | 0.09 | 0.24 |
| 16 | 0.94 | 0.88 | 0.97 | 0.00 | 0.00 | 0.46 | 0.06 | 0.03 | 0.12 |  | 0.81 | 0.42 | 0.94 | 0.09 | 0.00 | 0.47 | 0.10 | 0.06 | 0.18 |
| 17 | 0.35 | 0.00 | 0.80 | 0.31 | 0.00 | 0.67 | 0.34 | 0.18 | 0.58 |  | 0.51 | 0.14 | 0.90 | 0.35 | 0.00 | 0.70 | 0.14 | 0.08 | 0.24 |
| 18 | 0.92 | 0.84 | 0.96 | 0.00 | 0.00 | 0.31 | 0.08 | 0.04 | 0.16 |  | 0.48 | 0.02 | 0.89 | 0.34 | 0.00 | 0.74 | 0.18 | 0.10 | 0.34 |
| 19 | 0.69 | 0.10 | 0.91 | 0.13 | 0.00 | 0.64 | 0.18 | 0.09 | 0.38 |  | 0.76 | 0.03 | 0.87 | 0.00 | 0.00 | 0.67 | 0.24 | 0.13 | 0.43 |
| 20-69 | 0.92 | 0.61 | 0.94 | 0.00 | 0.00 | 0.31 | 0.08 | 0.06 | 0.13 |  | 0.91 | 0.59 | 0.94 | 0.00 | 0.00 | 0.32 | 0.06 | 0.09 | 0.12 |
| East-Asian males with high parental education | | | | | | | | | |  | East-Asian females with high parental education | | | | | | | | |
| 1 | 0.34 | 0.04 | 0.84 | 0.56 | 0.05 | 0.83 | 0.11 | 0.05 | 0.23 |  | 0.03 | 0.00 | 0.43 | 0.81 | 0.41 | 0.91 | 0.16 | 0.08 | 0.33 |
| 2 | 0.57 | 0.07 | 0.95 | 0.33 | 0.00 | 0.80 | 0.10 | 0.05 | 0.23 |  | 0.32 | 0.04 | 0.96 | 0.62 | 0.00 | 0.87 | 0.06 | 0.03 | 0.14 |
| 3 | 0.35 | 0.14 | 0.76 | 0.59 | 0.18 | 0.80 | 0.06 | 0.03 | 0.11 |  | 0.56 | 0.16 | 0.94 | 0.34 | 0.00 | 0.72 | 0.10 | 0.05 | 0.20 |
| 4 | 0.18 | 0.00 | 0.62 | 0.76 | 0.31 | 0.92 | 0.06 | 0.03 | 0.14 |  | 0.42 | 0.19 | 0.90 | 0.56 | 0.09 | 0.79 | 0.02 | 0.01 | 0.04 |
| 5 | 0.45 | 0.21 | 0.92 | 0.52 | 0.04 | 0.75 | 0.03 | 0.02 | 0.07 |  | 0.93 | 0.44 | 0.96 | 0.00 | 0.00 | 0.48 | 0.07 | 0.04 | 0.15 |
| 6 | 0.62 | 0.30 | 0.97 | 0.34 | 0.00 | 0.67 | 0.04 | 0.02 | 0.08 |  | 0.20 | 0.03 | 0.50 | 0.75 | 0.44 | 0.89 | 0.05 | 0.03 | 0.12 |
| 7 | 0.61 | 0.28 | 0.94 | 0.30 | 0.00 | 0.62 | 0.09 | 0.05 | 0.17 |  | 0.66 | 0.29 | 0.95 | 0.26 | 0.00 | 0.63 | 0.08 | 0.05 | 0.14 |
| 8 | 0.77 | 0.45 | 0.98 | 0.21 | 0.00 | 0.53 | 0.02 | 0.01 | 0.04 |  | 0.25 | 0.09 | 0.54 | 0.70 | 0.40 | 0.85 | 0.05 | 0.03 | 0.09 |
| 9 | 0.90 | 0.51 | 0.96 | 0.03 | 0.00 | 0.41 | 0.07 | 0.04 | 0.13 |  | 0.91 | 0.56 | 0.95 | 0.00 | 0.00 | 0.35 | 0.09 | 0.05 | 0.16 |
| 10 | 0.38 | 0.18 | 0.73 | 0.57 | 0.21 | 0.77 | 0.05 | 0.03 | 0.09 |  | 0.70 | 0.37 | 0.96 | 0.24 | 0.00 | 0.57 | 0.06 | 0.04 | 0.09 |
| 11 | 0.72 | 0.30 | 0.93 | 0.16 | 0.00 | 0.58 | 0.11 | 0.07 | 0.19 |  | 0.94 | 0.90 | 0.96 | 0.00 | 0.00 | 0.19 | 0.06 | 0.04 | 0.10 |
| 12 | 0.33 | 0.15 | 0.68 | 0.64 | 0.28 | 0.81 | 0.04 | 0.02 | 0.06 |  | 0.45 | 0.21 | 0.92 | 0.50 | 0.03 | 0.74 | 0.05 | 0.03 | 0.08 |
| 13 | 0.16 | 0.00 | 0.49 | 0.75 | 0.42 | 0.90 | 0.09 | 0.05 | 0.15 |  | 0.82 | 0.48 | 0.94 | 0.10 | 0.00 | 0.44 | 0.08 | 0.05 | 0.13 |
| 14 | 0.73 | 0.36 | 0.97 | 0.23 | 0.00 | 0.60 | 0.04 | 0.03 | 0.07 |  | 0.63 | 0.27 | 0.93 | 0.26 | 0.00 | 0.61 | 0.11 | 0.06 | 0.18 |
| 15 | 0.33 | 0.06 | 0.95 | 0.61 | 0.00 | 0.86 | 0.07 | 0.04 | 0.13 |  | 0.93 | 0.68 | 0.96 | 0.00 | 0.00 | 0.25 | 0.07 | 0.04 | 0.12 |
| 16 | 0.30 | 0.03 | 0.76 | 0.58 | 0.12 | 0.82 | 0.12 | 0.07 | 0.22 |  | 0.79 | 0.36 | 0.93 | 0.10 | 0.00 | 0.53 | 0.11 | 0.06 | 0.20 |
| 17 | 0.44 | 0.00 | 0.91 | 0.39 | 0.00 | 0.84 | 0.17 | 0.09 | 0.35 |  | 0.78 | 0.33 | 0.97 | 0.17 | 0.00 | 0.63 | 0.05 | 0.03 | 0.10 |
| 18 | 0.38 | 0.00 | 0.92 | 0.49 | 0.00 | 0.86 | 0.14 | 0.07 | 0.28 |  | 0.74 | 0.34 | 0.97 | 0.20 | 0.00 | 0.60 | 0.06 | 0.03 | 0.13 |
| 19 | 0.32 | 0.00 | 0.94 | 0.55 | 0.00 | 0.91 | 0.13 | 0.06 | 0.33 |  | 0.81 | 0.38 | 0.97 | 0.14 | 0.00 | 0.56 | 0.06 | 0.03 | 0.12 |
| 20-69 | 0.58 | 0.33 | 0.94 | 0.35 | 0.00 | 0.61 | 0.07 | 0.05 | 0.09 |  | 0.89 | 0.56 | 0.93 | 0.03 | 0.00 | 0.36 | 0.09 | 0.07 | 0.11 |

LL: lower limit of confidence interval; UL: upper limit

Supplementary table 6. Regression coefficients from meta-regression analyses of the aggregate-level data of standardized variance components of height by parental education (reference category: low parental education)

|  | Intermediate parental education | High parental education |
| --- | --- | --- |
| Males |  |  |
| a^2^ | 0.101 (0.027, 0.176) | 0.054 (-0.023, 0.130) |
| c^2^ | -0.097 (-0.168, -0.025) | -0.049 (-0.122, 0.025) |
| e^2^ | -0.002 (-0.014, 0.009) | 0.000 (-0.011, 0.012) |
| Females |  |  |
| a^2^ | 0.030 (-0.036, 0.097) | 0.053 (-0.013, 0.119) |
| c^2^ | -0.038 (-0.097, 0.021) | -0.041 (-0.100, 0.018) |
| e^2^ | -0.004 (-0.016, 0.007) | -0.005 (-0.017, 0.007) |
| Both sexes |  |  |
| a^2^ | 0.068 (0.017, 0.118) | 0.057 (0.005, 0.108) |
| c^2^ | -0.068 (-0.115, -0.021) | -0.049 (-0.097, -0.001) |
| e^2^ | -0.003 (-0.011, 0.005) | -0.002 (-0.010, 0.006) |

():95% Confidence Intervals
